# Imidazole-modified G-quadruplex DNA as metal-triggered peroxidase†
†Electronic supplementary information (ESI) available: Analytical data, native ESI-MS, ABTS assay, synthesis and MD simulation. See DOI: 10.1039/c8sc05020a


**DOI:** 10.1039/c8sc05020a

**Published:** 2019-01-07

**Authors:** Philip M. Punt, Guido H. Clever

**Affiliations:** a TU Dortmund University , Faculty for Chemistry and Chemical Biology , Otto-Hahn-Str. 6 , 44227 Dortmund , Germany . Email: guido.clever@tu-dortmund.de

## Abstract

Four imidazoles, serving as metalloprotein-inspired ligands for complexing a range of transition metal cations, were incorporated into tetramolecular G-quadruplex DNA structures. Modified quadruplexes were found to complex Cu(ii), Ni(ii), Zn(ii) and Co(ii) in a 1 : 1 ratio with unprecedented strong thermal stabilizations of up to Δ*T*_1/2_ = +51 °C. Furthermore, addition of Cu(ii) was found to lead to extraordinarily fast G-quadruplex association rates with *k*_on_ values being ∼100 times higher compared to unmodified G-quadruplexes. This is ascribed to a template effect of Cu(ii), preorganizing the four single strands *via* coordination, followed by rapid formation of hydrogen-bonded G-quartets. Native electrospray ionization mass spectrometry (ESI), coupled with trapped ion-mobility spectrometry (timsTOF), supports the proposed 1 : 1 G-quadruplex-metal complexes and could further disclose their ability to bind the iron–porphyrin complex hemin in a 1 : 1 stoichiometry. DNA sequence design allowed us to equip this G-quadruplex-hemin complex, known to function as a horseradish peroxidase mimic, with a metal-dependent trigger. A competitive screen of transition metals revealed a high selectivity for Cu(ii), even in mixtures of several divalent metal cations. Once formed, the Cu(ii)-carrying DNAzyme was shown to be preserved in the presence of EDTA, attributed to its remarkable kinetic stability. Stimuli-responsive G-quadruplexes promise application in DNAzymes with switchable activity, adaptive sensors and dynamic DNA origami constructs.

## Abstract 2

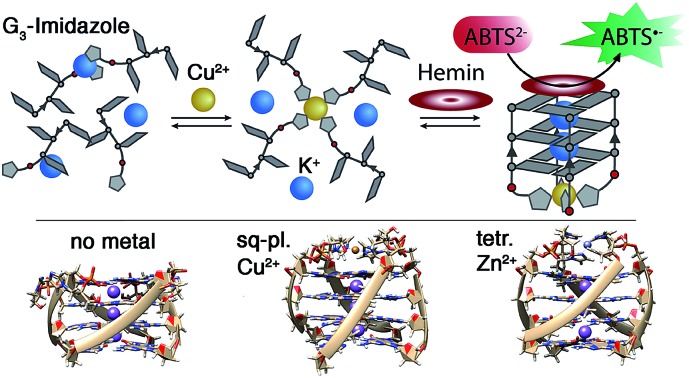
Imidazole-modified G-quadruplex DNA binds transition metals Cu(ii), Zn(ii), Ni(ii) and Co(ii). Metal-triggered association with hemin generates a stimuli-responsive peroxidase mimic.

## Introduction

Depending on base sequence and solution conditions, DNA can fold into different secondary structures including canonical double strands as well as triplexes, cytosine-rich i-motifs or G-quadruplexes.^1^ The latter ones assemble from guanine-rich sequences to form π-stacked G-tetrads, held together *via* circular Hoogsteen base pairing patterns containing central monovalent cations, often Na^+^ and K^+^. Structurally, G-quadruplexes show a higher diversity than one might expect. While the G-tetrads offer only a small structural variety (*anti/syn* glycosidic angles), the quadruplexes' overall diversity arises from a choice of different strand molecularities (tetra-, tri-, bi- and unimolecular), orientations (parallel or antiparallel) and loop lengths.

In recent years, G-quadruplexes moved into the focus of research in chemical biology for their ubiquitous presence in the human genome and their potential relevance for diseases such as HIV and cancer.^2^–^4^ On a parallel time scale, the field of DNA nano-technology emerged, including the sub-discipline of metal-mediated base pairing which studies the replacement of the regular Watson–Crick hydrogen bonding scheme by metal coordination. While few examples comprise the bridging of natural DNA bases by metal cations (such as in T–Hg^2+^–T and C–Ag^+^–C), a large variety of artificial base surrogates was introduced, allowing to serve as ligands for a wider range of transition metal cations.^5^–^7^ Eventually, this development lead to the design of longer, mixed metal arrays that facilitated a regioselective positioning of different metals to construct nano-wires.^8^,^9^ Until recently, however, most reported examples have featured duplex DNA and only few publications reported on metal-mediated base interaction in higher secondary structures such as triplex DNA, three way junctions, i-motifs and G-quadruplexes.^10^–^14^ In 2013, we reported the first metal-mediated G-quadruplexes where one G-tetrad was replaced by four pyridine modifications, allowing the complexation of Cu(ii) and Ni(ii).^15^ This system was exploited as Cu(ii)-sensitive topology-switch in unimolecular G-quadruplexes, as Cu(ii)-activated thrombin inhibitor and for the design of EPR-based (Electron Paramagnetic Resonance) molecular rulers.^16^–^18^

In a different branch of DNA nanotechnology, pre-established metal complexes were incorporated into chiral DNA scaffolds for the design of artificial DNAzymes.^19^,^20^ Examples include systems capable of DNA ligation or hydrolysis, enantioselective *syn*-hydration of enones, Diels–Alder reactions, Michael additions, and the horseradish peroxidase-mimicking hemin-G-quadruplex DNAzyme, one of the most intensively explored examples.^19^–^24^ It was shown that the latter system can catalyse the oxidation of organic substrates to fluorophores or chromophores. Also, it catalyses biologically relevant oxidations of thiols and NADH.^25^,^26^ Applications in DNA nanotechnology include the design of logic gates, heavy metal detection and cholesterol diagnostics.^27^–^29^

Here we report the first covalent introduction of an imidazole-modified nucleobase surrogate into tetramolecular G-quadruplexes. Our study is inspired by the ubiquitous involvement of the amino acid histidine in the coordination of transition metal cations in numerous metalloproteins. Different biorelevant metal cations were found to be complexed by these modified G-quadruplex structures resulting in unprecedented high thermal stabilizations and highly accelerated association rates, culminating in the design of a Cu(ii)-dependent horseradish peroxidase-mimicking DNAzyme.

## Results and discussion

**Fig. 1:**
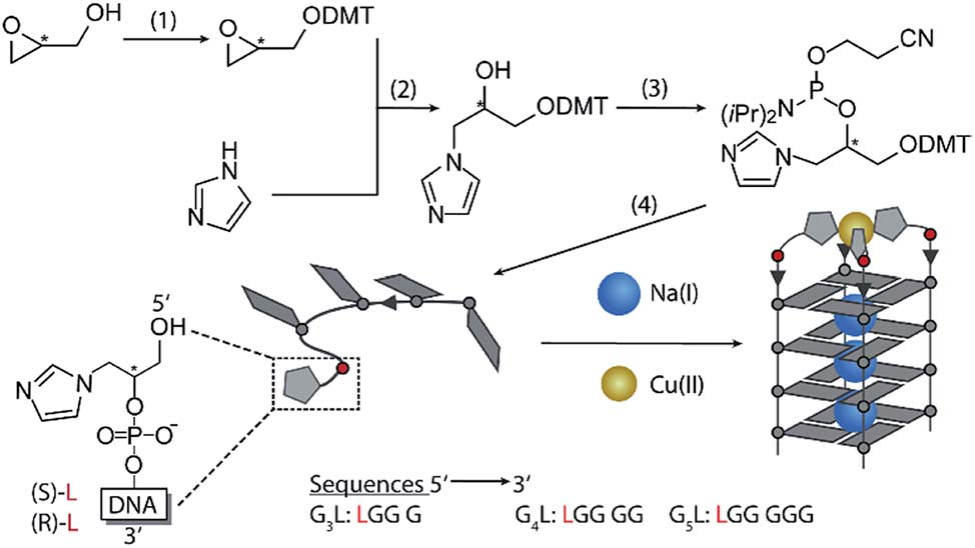
Synthesis of the phosphoramidites. (1) (*R/S*)-glycidol, Et_3_N, DMT-Cl in CH_2_Cl_2_ at room temperature (rt). (2) DMT-(*R/S*)-glycidol, imidazole in dioxane at 80 °C. (3) DMT-imidazole, DIPEA, CEDIP-Cl in CH_2_Cl_2_ at rt. (4) Solid phase synthesis. Bottom: schematic Cu(ii)-mediated G-quadruplex formation.

Inspired by the amino acid histidine and its omnipresent involvement in the coordination environments of metallo-enzymes, we aimed to incorporate imidazole derivatives into G-quadruplex DNA structures. The imidazole-based ligand **L** was introduced as GNA (glycol nucleic acid) building block enabling internal as well as terminal incorporation (Fig. 1). To access the phosphoramidites needed for solid phase synthesis, literature procedures were modified.^30^,^31^ An initial nucleophilic ring-opening of DMT-protected (*R/S*)-glycidol was followed by a phosphorylation reaction to access the required phosphoramidite building blocks, each as *R*- (**L**^***R***^) and *S*- (**L**^***S***^) enantiomers. DNA solid phase synthesis was performed according to standard protocols.^15^ Coupling times for **L** were extended to maximize reaction efficiencies, which were usually > 99% per coupling. After solid phase synthesis, DNA samples were deprotected in aqueous ammonia at 55 °C and purified ‘DMT-on’ using reversed-phase HPLC. After purification, the DMT-group was removed using C_18_ cartridges and aqueous TFA.

**Fig. 2:**
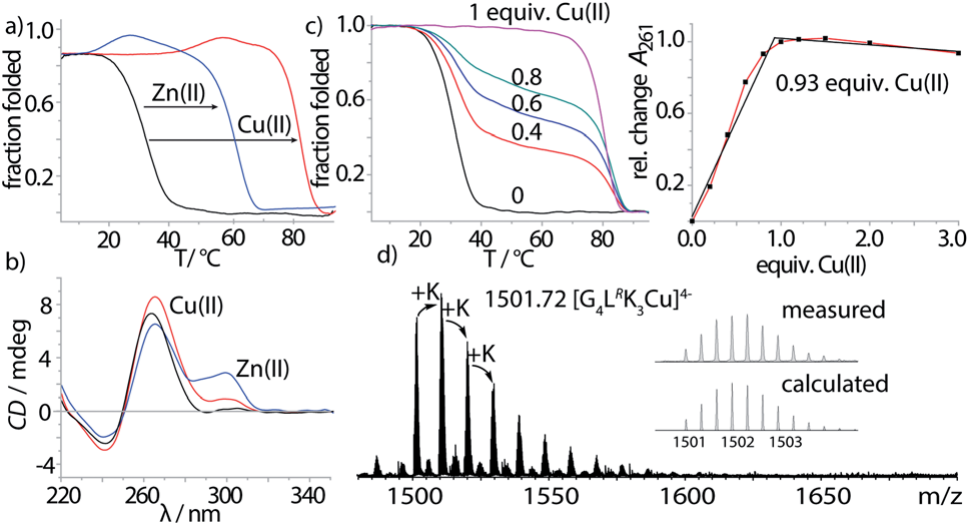
(a) Thermal denaturation experiments of G_4_**L**^***R***^ plus different divalent metal cations, recorded at 295 nm, (b) CD spectra of G_4_**L**^***R***^ before and after addition of Cu(ii) and Zn(ii), (c) Cu(ii) titration into G_4_**L**^***R***^ monitored by melting profile determination (left) and absorption at 261 nm, and (d) native ESI-MS of G_4_**L**^***R***^ in presence of Cu(ii).

Properties of the imidazole-modified G-quadruplexes were investigated using UV-Vis-based melting experiments, thermal difference spectra (TDS), circular dichroism (CD) spectroscopy and high-resolution ESI mass spectrometry. In case of melting experiments, the temperature-dependent change of absorption at 295 nm was observed, characteristic for G-quadruplex denaturation/renaturation.^32^,^33^ To prove whether the imidazole modification would still allow G-quadruplex formation, both diastereomers of the tetramolecular G-quadruplexes G_4_**L**^***R/S***^ and G_5_**L**^***R/S***^, containing four and five consecutive guanine residues followed by the 5′-imidazole modification, were investigated. G-quadruplex formation could be observed in the CD spectra (Fig. 2b, ESI Fig. 13–15†), with a characteristic positive Cotton effect at ∼262 nm consistent with a parallel topology.^34^ In thermal denaturation experiments for G_4_**L**^***R/S***^, significantly lower melting temperatures (*T*_1/2_(G_4_**L**^***R***^) = 32 °C, *T*_1/2_ (G_4_**L**^***S***^) = 31 °C) as compared to G_5_**L**^***R/S***^ (*T*_1/2_(G_5_**L**^***R***^) = 77 °C, *T*_1/2_ (G_5_**L**^***S***^) = 78 °C) were observed, owing to the one G-tetrad shorter sequence in G_4_**L**^***R/S***^ (Fig. 2a, Table 1).

**Table 1:** Thermal stabilities expressed as melting temperature *T*_1/2_ for G_3_**L**^***R/S***^, G_4_**L**^***R*/*S***^ and G_5_**L**^***R/S***^ in absence and presence of Cu(ii), Ni(ii), Zn(ii) and Co(ii) (in brackets, thermal stabilizations Δ*T*_1/2_ after metal addition are given)

Thermal stability *T*_1/2_ (Δ*T*_1/2_)/°C					
	—	Cu(ii)	Ni(ii)	Zn(ii)	Co(ii)
G_3_**L**^***S***^	—	40	n. d.	—	—
G_3_**L**^***R***^	—	38	n. d.	—	—
G_4_**L**^***S***^	31	79 (+48)	77 (+46)	54 (+23)	64 (+33)
G_4_**L**^***R***^	32	83 (+51)	81 (+49)	61 (+29)	73 (+41)
G_5_**L**^***S***^	78	>95	>95	87 (+9)	>95
G_5_**L**^***R***^	77	>95	>95	86 (+9)	>95

After having established the formation of parallel G-quadruplexes, metal complexation was investigated. When 1 equiv. of Cu(ii) was added to G_4_**L**^***R***^, an unprecedented high stabilization towards thermal denaturation of Δ*T*_1/2_ = +51 °C was observed, while retaining the parallel topology as indicated by CD spectroscopy (Fig. 2b). In addition, a new maximum at ∼295 nm appeared which was attributed to a previously reported flipping of the 3′-guanine residues.^17^ In case of already much more stable G_5_**L**^***R***^, Cu(ii) addition resulted in thermal stabilities > 95 °C and no denaturation could be observed anymore. For the isomeric sequence G_4_**L**^***S***^, thermal stabilization was significantly lower (Δ*T*_1/2_ = +48) as compared to its diastereomer G_4_**L**^***R***^, indicating that the configuration of **L** plays an important role when it comes to metal complexation. This is in contrast to the metal-free G-quadruplex, where the modification's stereochemistry had only a neglectable influence. UV-Vis spectroscopy-based titrations of the ligand-modified quadruplex with Cu(ii) cations showed to be in agreement with a proposed 1 : 1 stoichiometry (Fig. 2c) of Cu(ii) and G-quadruplex. Interestingly, when thermal denaturation studies of G_4_**L**^***R***^ were performed with Cu(ii)-unsaturated systems, two clear transitions were observed at *T*_1/2_ = 32 °C and *T*_1/2_ = 83 °C, corresponding to Cu(ii)-free and -bound G-quadruplex, respectively. Finally, evidence for formation of the proposed complex was obtained by native ESI mass spectrometry. To determine whether a G-quadruplex retains its native state in the gas phase, two observables can be followed. First, if the secondary structure is destroyed, single stranded DNA instead of a tetramer would be observed. Second, in their native state G_*n*_-quadruplexes bind n-1 monovalent cations in their central pockets (with *n* = number of G-tetrads). On top, ESI mass spectrometry from electrolyte-containing solutions always gives rise to series of unspecific adducts with sodium or potassium cations. For fully denatured species, a statistical distribution of adducts starting with zero cations would be observed while for a native, folded species a distribution is observed starting with n-1 explicitly bound cations.^35^ In case of Cu(ii)-bound G_4_**L**^***R***^, one main species [(G_4_**L**^***R***^)_4_K_3_Cu]^4–^ corresponding to a folded G-quadruplex with three potassium ions and a single Cu(ii) was observed as the most abundant and first peak of a cluster series, followed by further unspecific K^+^-adducts (Fig. 2d).

Next, the sample was subjected to trapped ion mobility time-of-flight mass spectrometry (timsTOF). With this method, the gas-phase conformational distribution of ionic molecules or supramolecular objects can be determined by measuring their relative mobility *K*_0_ when pushed against a variable electric field by a constant stream of collision gas. Large ions elute faster, while smaller ions remain longer trapped. Mobilities can be converted to collisional cross sections (CCS) by applying the Mason-Schamp equation (details see ESI†). For Cu(ii)-bound G_4_**L**^***R***^, very sharp ion-mobility distributions could be measured corresponding to a CCS of 793 Å^2^, being in the same range as similar, but unmodified tetramolecular G-quadruplexes and indicating a compact, well-defined 3-dimensional structure (Fig. 5, ESI Fig. 26†).^36^

To evaluate the scope of metal complexation, further transition metal cations were screened. Ni(ii), Zn(ii) and Co(ii) were found to bind to the modified G-quadruplexes as shown by strong thermal stabilizations (see Table 1) while retaining the parallel topology (ESI Fig. 14 and 15†). Noteworthy is the successful binding of Zn(ii), for which only this year a first example for a specific metal-mediated base pair in a DNA double strand was reported by Müller *et al.*^37^ To probe if the thermal stabilization was a reversible process, EDTA was added to the G-quadruplex-metal complexes. For Zn(ii) and Co(ii), EDTA addition led to an immediate reversal of the metal-induced thermal stability but in case of Cu(ii) and Ni(ii) no change was observed, indicating that EDTA is hindered of readily removing the bound Ni(ii) and Cu(ii) cations. This is attributed to a kinetically trapped state since no metal-mediated stabilisation was observed for Cu(ii) and Ni(ii) if EDTA was added prior to G-quadruplex formation and metal addition.1
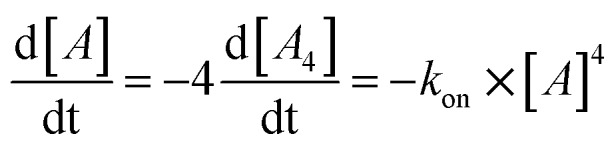



**Fig. 3:**
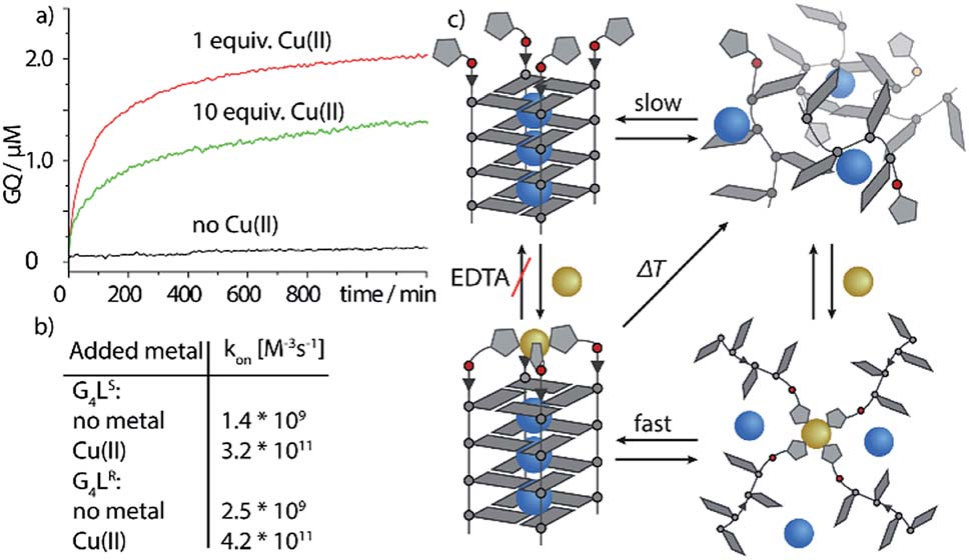
Kinetic studies of G-quadruplex assembly. (a) CD-monitored association of G_4_**L*^s^*** at 7 °C and 8 μM single strand (black) in absence of Cu(ii), (red) with 1 equiv. Cu(ii) and (green) with 10 equiv. Cu(ii). (b) Calculated *k*_on_ values using eqn (1) and (c) proposed metal-mediated G-quadruplex association model.

Next, we followed the association of G_4_**L**^***R/S***^ from its single strand components over time, indicated by the CD spectral change at 262 nm, at 7 °C (Fig. 3, ESI Fig. 22†). In absence of transition metals, the typical slow association was observed, stagnating without ever reaching full conversion after days. Association rate constant *k*_on_ was calculated using eqn (1) and was in a similar range to unmodified tetramolecular DNA G-quadruplexes reported in the literature (*k*_on_(G_4_**L**^***R***^) = 2.5 × 10^9^ M^–3^ s^–1^; *k*_on_(G_4_**L**^***S***^) = 1.4 × 10^9^ M^–3^ s^–1^).^38^,^39^ After addition of 1 equiv. Zn(ii), for G_4_**L**^***R***^ a 10-fold accelerated association rate (*k*_on_(G_4_**L**^***R***^-Zn) = 1.9 × 10^10^ M^–3^ s^–1^) was determined while for G_4_**L**^***S***^ only a weak acceleration could be observed (*k*_on_(G_4_**L**^***s***^-Zn) = 3.3 × 10^9^ M^–3^ s^–1^). When Cu(ii) was added, however, G-quadruplex association was enormously accelerated with a half-association time of only 10 min and full G-quadruplex formation was observed within hours. To calculate *k*_on_ for the metal containing G-quadruplexes, fourth-order kinetics were assumed as plausible model,^38^ based on the encounter of four individual DNA strands of which one, at a given Cu : DNA ratio of 1 : 4, brings in a rapidly precoordinated Cu(ii) cation. The correspondingly calculated values *k*_on_(G_4_**L**^***R***^-Cu) = 4.2 × 10^11^ M^–3^ s^–1^ and *K*_on_(G_4_**L**^***S***^-Cu) = 3.2 × 10^11^ M^–3^ s^–1^ reflect a 100-fold acceleration as compared to the metal free sample (Fig. 3a and b). Our hypothesis for the accelerated association is based on a very fast coordination of the imidazoles of four strands to Cu(ii), yielding an intermediate complex. This precoordinated species then quickly folds to the corresponding G-quadruplex under formation of Hoogsteen hydrogen bonds and incorporation of three sodium ions (Fig. 3c). Interestingly, when association kinetics were determined in presence of a tenfold excess of Cu(ii) cations, the reaction was becoming slower again (*k*_on_(G_4_**L**^***R***^-Cu) = 1.8 × 10^11^ M^–3^ s^–1^ and *K*_on_(G_4_**L**^***S***^-Cu) = 1.3 × 10^11^ M^–3^ s^–1^), as observed in CD (Fig. 3a) as well as UV-Vis spectroscopy experiments (ESI†). In light of the proposed model, we explain this with saturation of a majority of single strand imidazoles with coordinated Cu(ii) cations, rendering an encounter of four DNA strands around a single Cu(ii) less likely.

**Fig. 4:**
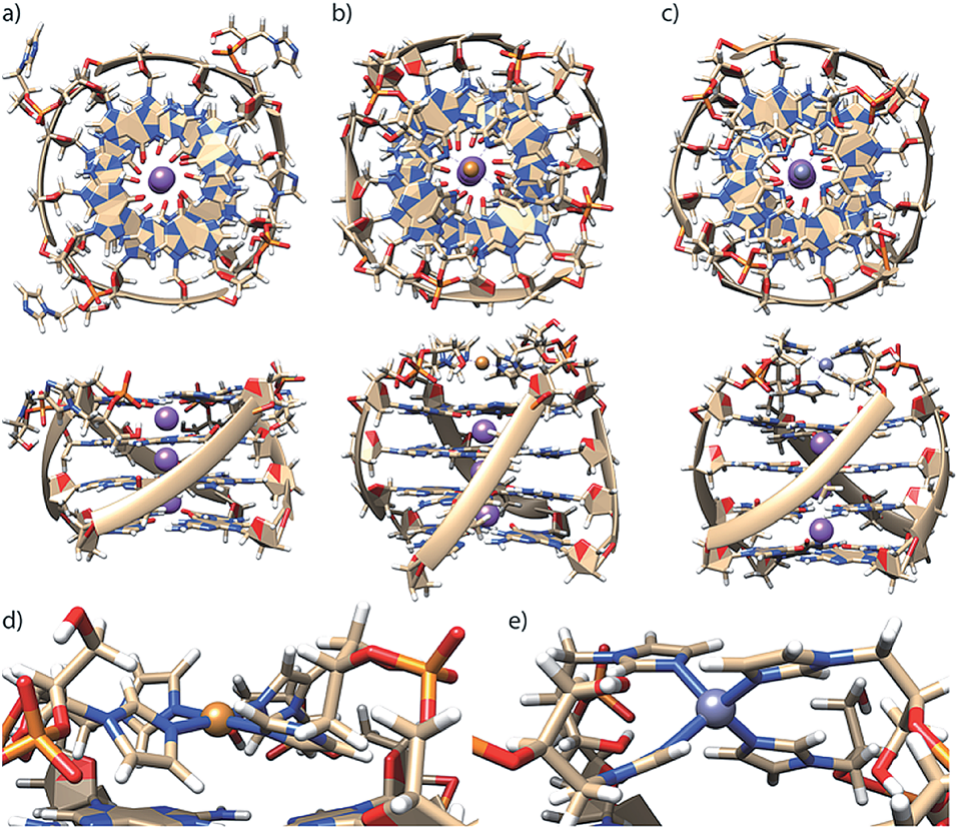
Molecular dynamics simulations of G_4_**L**^***R***^ in (a) metal-unbound state, in complex with (b) Cu(ii) and (c) Zn(ii). Coordination geometry details for (d) Cu(ii) and (e) Zn(ii). Simulations were performed in TIP3P water on a 50 ns timescale.

To better understand how G_4_**L**^***R/S***^ is able to accommodate both Cu(ii) and Zn(ii), since the former usually prefers square-planar and the latter tetrahedral coordination, MD simulations were performed using a bonded model for metal interactions (Fig. 4) according to a previously published protocol.^16^ In absence of Zn(ii) and Cu(ii), the modelled structure of G_4_**L**^***R***^ reveals that the imidazole ligands interact with the groves (Fig. 4a). This is in contrast to our previous observation for pyridine-carrying tetramolecular quadruplexes, where π-stacking of the aromatic modifications with the 3′-terminal G-tetrad was proposed based on MD simulations and melting studies.^17^ Interestingly, MD simulation of diastereomer G_4_**L**^***S***^ indeed found the ligand stacking on top of the 3′-G-tetrad. Subsequent MD simulations where performed in complex with Cu(ii) and Zn(ii), respectively, showing that the ligand was just long enough to facilitate a square-planar coordination for Cu(ii) (Fig. 4b and d) and a tetrahedral coordination for Zn(ii) (Fig. 4c and e).

**Fig. 5:**
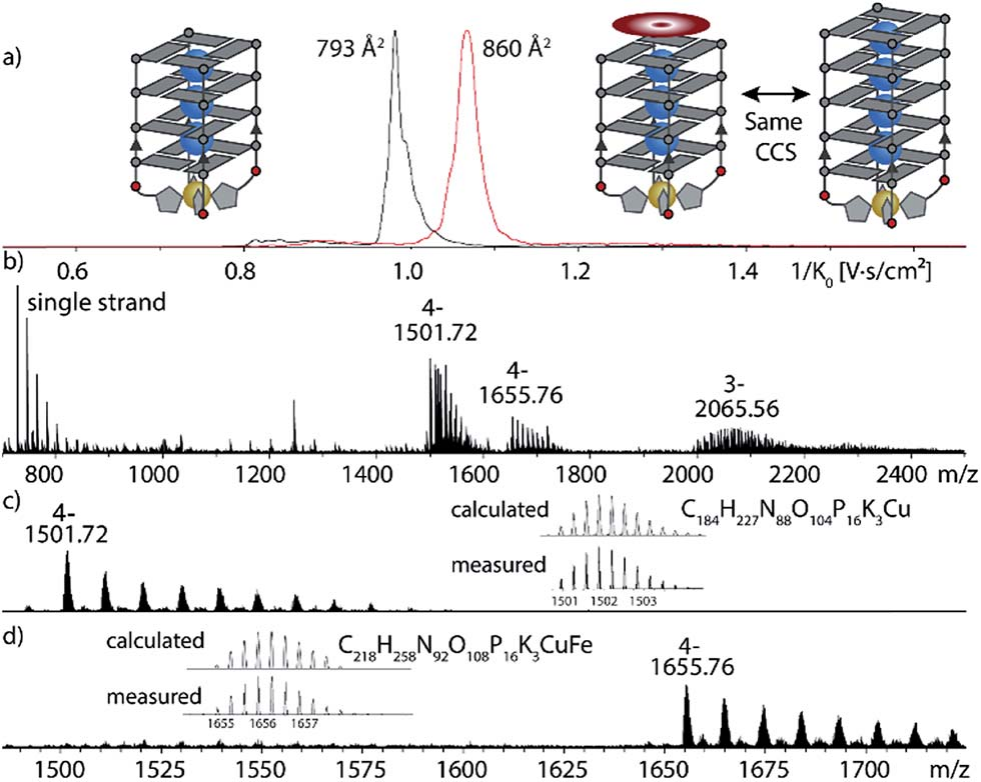
Native ESI-MS and trapped ion-mobility time-of-flight (timsTOF) experiments of G_4_**L**^***R***^ in complex with Cu(ii) and hemin. (a) Ion-mobilities and corresponding collisional cross sections; (b) full ESI-MS; (c) and (d) ion-mobility-extracted mass spectra for [(G_4_**L**^***R***^)_4_K_3_Cu]^4–^ and [(G_4_**L**^***R***^)_4_K_3_Cu-hemin]^4–^ hemin.

After exploring the scope of metal binding, we set out to exploit the tremendous thermal stabilization and accelerated assembly upon Cu(ii) addition to develop a switchable DNAzyme capable of mimicking the metalloenzyme horseradish peroxidase. As a crucial prerequisite for this purpose, the modified G-quadruplex has to maintain the capacity for binding the iron–porphyrin complex hemin. ESI-MS investigations could show that the modified G-quadruplex was indeed able to bind one molecule of hemin, as two main signals corresponding to [(G_4_**L**^***R***^)_4_K_3_Cu]^4–^ and [(G_4_**L**^***R***^)_4_K_3_Cu-hemin]^4–^ were observed (Fig. 5). No signal corresponding to a two-hemin adduct could be determined. In addition, ion-mobility experiments showed two well separated signals corresponding to [(G_4_**L**^***R***^)_4_K_3_Cu]^4–^ (793 Å^2^) and [(G_4_**L**^***R***^)_4_K_3_Cu-hemin]^4–^ with a significant larger CCS = 860 Å^2^. Noteworthy, the CCS increase of the hemin adduct exactly corresponded to the addition of one G-tetrad as observed for [(G_5_**L**^***R***^)_4_K_4_]^4–^ (858, Å^2^, ESI Fig. 27 and 28†).

As a readout for peroxidase-activity, the oxidation of ABTS with H_2_O_2_ to the strongly green ABTS-radical (*A*_max_ = 414 nm) was performed. To quantify the catalytic activity, the initial rate (*V*_0_) was determined, defined as the concentration of the ABTS radical as function of time. First experiments showed that G_4_**L**^***S***^-hemin indeed catalysed the oxidization of ABTS (*V*_0_ = 390 nM s^–1^), however, even in absence of Cu(ii) G_4_**L**^***S***^ was already stable at 25 °C (*T*_1/2_ = 31 °C (*R*), 32 °C (*S*)), hemin binding further increased the thermal stability (G_4_**L**^***S***^-hemin *T*_1/2_ = 45 °C, ΔT_1/2_ = +14 °C) and thus Cu(ii) addition only resulted in a small activity increase (*V*_*0*_ = 523 nM s^–1^, ESI Fig. 24†). To destabilize the system, the concentration of NaCl was reduced to 1 mM but still G_4_**L**^***S***^-hemin was forming in absence of Cu(ii) (ESI Fig. 7†). However, when the number of G-tetrads was reduced to three, the resulting G-quadruplex G_3_**L**^***R/S***^ was extremely unstable, and no G-quadruplex formation could be observed at 7 °C by CD (ESI Fig. 16†). While addition of Zn(ii) and Co(ii) could not trigger G-quadruplex formation, addition of either Cu(ii) or Ni(ii) lead to the formation a parallel stranded G-quadruplex. In case of Ni(ii), G-quadruplex formation was not complete as indicated by CD spectroscopy. In case of Cu(ii), quadruplex formation was smooth, thus enabling the design of a switchable peroxidase mimic.

**Fig. 6:**
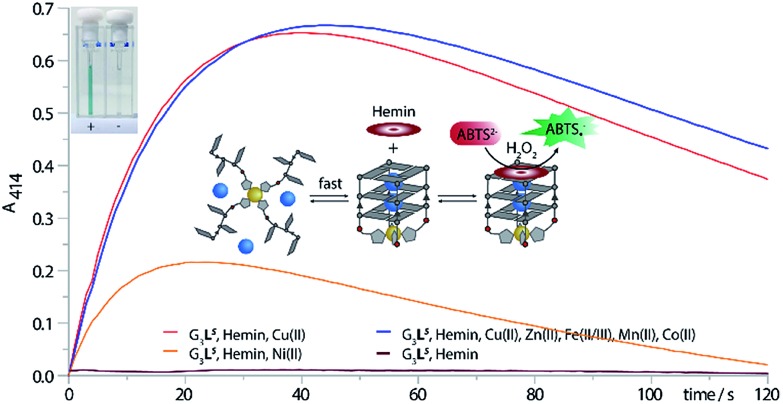
ABTS assay. The assay was performed with 0.625 μM G-quadruplex, 0.625 μM transition metal, 0.5 μM hemin, 50 mM NaCl, 5 mM HEPES pH8, 2 mM ABTS and 0.5 mM H_2_O_2_. Top left: (+) cuvette containing G_3_**L**^***S***^, Cu(ii) and hemin and (–) cuvette containing only G_3_**L**^***S***^ and hemin.

As expected, when the oxidation of ABTS was performed only with G_3_**L**^***R***^ or G_3_**L**^***S***^ and hemin, no activity could be observed. Also after addition of various transition metal cations such as Fe(ii/iii), Zn(ii), Co(ii) or Mn(ii) no activity was observed. Only when G_3_**L**^***R***^ or G_3_**L**^***S***^ was formed in presence of either Cu(ii) or Ni(ii) and hemin, the active DNAzyme could form, showing a strong catalytic activity (Fig. 6, ESI Fig. 25†). In presence of Cu(ii), a significant higher initial rate was determined (*V*_0_(*R*) = 785 nM s^–1^, *V*_0_(*S*) = 615 nM s^–1^) than in presence of Ni(ii) (*V*_0_(*R*) = 387 nM s^–1^, *V*_0_(*S*) = 369 nM s^–1^) which was attributed to the incomplete G-quadruplex formation with Ni(ii). The DNAzyme was also shown to be stable after addition of up to 10 equiv. of EDTA and incubation at 25 °C for 1 h, attributed to a kinetically trapped metal complex, however an activity decrease (*V*_0_(*R*) = 281 nM s^–1^, *V*_0_(*S*) = 353 nM s^–1^) was observed. The decrease was mainly attributed to a general interference of EDTA in this assay which was further supported by time-dependent CD spectroscopy of G_3_**L**^***R***^ and G_3_**L**^***S***^ in presence of EDTA (ESI Fig. 17†) that was showing no signs of G-quadruplex decomposition after 1 h at 7 °C.^40^ Finally, when the DNAzyme was formed in presence of Cu(ii) in mixture with competing transition metals including Fe(ii/iiiIII), Mn(ii), Zn(ii) and Co(ii/iii), only a modest deceleration was observed for G_3_**L**^***R***^ (*V*_0_ = 712 nM s^–1^) while for G_3_**L**^***S***^ (*V*_0_ = 641 nM s^–1^; Fig. 6, blue line), even a small rate increase was observed. Hence, our system shows potential for Cu(ii) sensory applications in the presence of competing metal ions.

## Conclusions

The covalent introduction of imidazole ligands into tetramolecular G-quadruplex structures is reported. Modified G-quadruplexes were shown to complex several transition metal cations such as Cu(ii), Zn(ii), Co(ii) and Ni(ii). In case of Cu(ii), metal complexation lead to an unprecedented strong thermal stabilization and highly accelerated association rate which could be exploited to design a Cu(ii)-switchable peroxidase. Once formed, the peroxidase was shown to be stable in presence of EDTA, attributed to the formation of a kinetically trapped complex. Detailed mass spectrometric investigations could show that only one hemin was binding to the 3′-end, while the 5′-end was blocked by the formed metal complex. Further, trapped ion-mobility experiments indicated a significant increase of the collisional cross section (CCS) upon hemin binding from 793 to 860 Å^2^ which exactly corresponded to the CCS of a one G-tetrad larger G-quadruplex (858 Å^2^). The herein established switchable DNAzyme presents a novel approach to stimuli-responsive, functional oligonucleotide structures with potential application in diagnostic setups, triggered probes for chemical biology studies as well as dynamic DNA nano-technological devices.

## Conflicts of interest

There are no conflicts to declare.

## Supplementary Material

Supplementary information

Supplementary information

Supplementary information

Supplementary information

Supplementary information

Supplementary information

Supplementary information

Supplementary information

Supplementary information

Supplementary information

Supplementary information

Supplementary information

Supplementary information
