# Supplementary material for: Imidazole-modified G-quadruplex DNA as metal-triggered peroxidase
Source: Chem Sci. 2019 Jan 7;10(8):2513–8. doi: 10.1039/c8sc05020a (PMC6399679; doi:10.1039/c8sc05020a)
Supplement: Supplementary file 5 [file SC-010-C8SC05020A-s005.pdf]

Electronic Supplementary Material (ESI) for Chemical Science.
This journal is © The Royal Society of Chemistry 2019

Supporting Information

Imidazole-modified G-quadruplex DNA as Metal-triggered Peroxidase

Philip M. Punt and Guido H. Clever

Table of content

1 Synthesis of imidazole phosphoramidite 3
1.1 Synthesis of DMT protected glycidol (2) 3
1.2 DMT-protected glycidol-based imidazole nucleoside (3) 3
1.3 Synthesis of the phosphoramidite (4) (*R/S*) 4
2 DNA synthesis and purification 4
2.1 Oligonucleotides 4
2.2 Reversed phase purification 4
3 Thermal denaturation studies 4
3.1 UV-Vis spectroscopy 4
3.2 G_5L^R 5
3.3 G_5L^S 5
3.4 G_4L^R 6
3.5 G_4L^S 7
3.6 G_3L^R and G_3L^S in presence of 1 equiv. Cu(II) 8
3.7 G_3L^R and G_3L^S in presence of 1 equiv. Cu(II) and 10 equiv. EDTA 8
3.8 G_4L^S stability screen 9
3.9 G_4L^S in presence of 10 equiv. EDTA 9
3.10 G_4L^R in presence of 10 equiv. EDTA 10
4 Thermal difference spectra (TDS) 10
4.1 G_5L^{RS} 10
4.2 G_4L^R 11
4.3 G_4L^S 11
5 Circular dichroism measurements 12

5.1 CD spectroscopy ... 12
5.2 G_5L^{RS} ... 12
5.3 G_4L^{R} ... 13
5.4 G_4L^{S} ... 13
5.5 G_3L^{R} and G_3L^{S} ... 14
5.6 Time-resolved CD-spectroscopy of G_3L^{R} and G_3L^{S} after EDTA addition ... 14
6 MD simulations ... 14
6.1 Partial charges (RESP) ... 15
7 ABTS assay ... 20
7.1 ABTS assay with G_4L^{S} ... 21
7.2 ABTS assay with G_3L^{S}. ... 21
8 Native ESI mass spectrometry ... 21
8.1 Sample preparation ... 21
8.2 Trapped Ion Mobility ESI Mass Spectrometry ... 21
8.3 Native ESI-MS of $G_4L^{R}Cu$ ... 23
8.4 Native ESI-MS of G_5L^{R} ... 23
8.5 Native ESI-MS of $G_5L^{S}Cu$ ... 24
8.6 Native ESI-MS of G_5L^{S} ... 24
8.7 Native ESI-MS of $G_5L^{S}Cu$ ... 25
9 Further analytical data ... 26
9.1 Analytical data of G_3L^{R} ... 27
9.2 Analytical data of G_3L^{S} ... 27
9.3 Analytical data of G_4L^{R} ... 28
9.4 Analytical data of G_4L^{S} ... 28
9.5 Analytical data of G_5L^{R} ... 29
9.6 Analytical data of G_5L^{S} ... 29

1 Synthesis of imidazole phosphoramidite

The synthetic route is based on two earlier publications dealing with the access of GNA based nucleotide derivatives.[1,2]

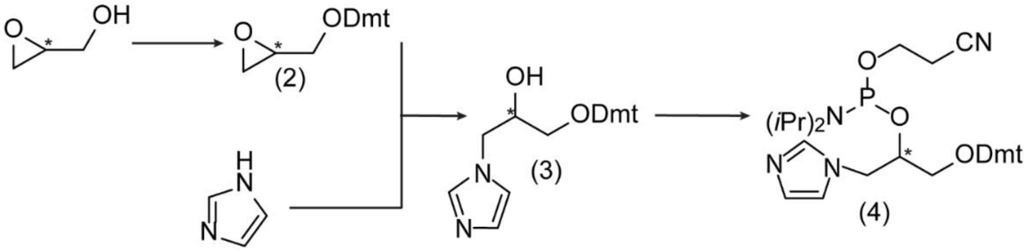

1.1 Synthesis of DMT protected glycidol (2)

Compound **2** was prepared according to modified literature procedures.[1] To a solution of glycidol (*R/S*) (0.5 mL, 7.5 mmol, 1.09 equiv.) in dry CH_2Cl_2 (17 mL) triethylamine (2.7 mL, 19.4 mmol, 2.8 equiv.) was added under argon. The solution was stirred for five minutes before DMT-chloride (2.35 g, 6.9 mmol, 1 equiv.) was slowly added and stirred for 16 h at 25 °C. The reaction mixture was washed with half saturated aq. $NaHCO_3$ (50 mL) and extracted with CH_2Cl_2 (3 × 30 mL). The combined organic layers were dried over $MgSO_4$ and the solvent was removed under reduced pressure to afford a dark red oil. The product was purified by column chromatography (cyclohexane/EtOAc/Et_3N, 99.5/0/0.5 → cyclohexane/EtOAc/Et_3N, 89.5/10/0.5) to afford the product (**2**) as a viscous colourless oil.

^{1}H-NMR (600 MHz, $CDCl_3$): σ (/ppm) = 2.63 (dd, 1H, CH_2), 2.78 (dd, 1H, CH_2), 3.13 (dd, 1H, CH_2), 3.32 (dd, 1H, CH_2), 3.79 (s, 6H, $-OCH_3$), 6.82 – 6.85 (m, 4H, CH), 7.20 – 7.23 (m, 1H, CH), 7.28 – 7. 31 (m, 2H, CH), 7.34 – 7.37 (m, 4H, CH), 7.46 – 7.48 (m, 2H, CH).

^{13}C-NMR (150 MHz): σ (/ppm) = 45.0, 51.5, 55.6, 64.9, 86.5, 113.5, 127.1, 128.2, 128.5, 130., 136.4, 145.1, 158.8.

**Chemical formula**:	$C_{24}H_{24}O_4$
**Molecular weight**:	376.45 g mol^{-1}
**Yield**:	2.05 g, 5.4 mmol, 79 %

1.2 DMT-protected glycidol-based imidazole nucleoside (3)

To a solution of imidazole (0.54 g, 8 mmol, 2 equiv.) in 1,4-dioxane (7.5 mL) a solution of (**2**) (*R/S*) in 1,4-dioxane (2.5 mL) was added. The reaction was stirred at 80 °C for 21 h until all starting material was consumed. The reaction mixture was washed with half saturated aq. $NaHCO_3$ (40 mL) and extracted with CH_2Cl_2 (3 × 30 mL). The combined organic layers were dried over $MgSO_4$ and the solvent was removed under reduced pressure to afford a yellow oil. The product was purified by flash chromatography ($CHCl_3$/MeOH/Et_3N, 99.5/0/0.5 → $CHCl_3$/MeOH/Et_3N, 89.5/10/0.5) to afford the product (**3**) as a white foamy solid.

^{1}H-NMR (600 MHz, $CDCl_3$): σ (/ppm) = 3.14 (dd, 1H, 6-CH_2); 3.22 (dd, 1H, 6-CH_2); 3.6 (s_{broad}, 1H, OH); 3.79 (s, 6H, 20-CH_3); 3.91 – 3.95 (m, 1H, 7-CH), 3.95 (dd, 1H, 8-CH_2); 4.08 (dd, 1H, 8-CH_2); 6.82 – 6.86 (m, 6H, 2-CH, 5-CH, 13-CH); 7.22-7.24 (m, 1H, 16-CH); 7.29-7.32 (m, 7H, 3-CH, 12-CH, 17-CH); 7.41-7.43 (m, 2H, 16-CH).

^{13}C-NMR (150 MHz): σ (/ppm) = 50.6, 55.6, 64.8, 70.5, 68.8, 113.6, 120.0, 127.3, 128.3, 128.4, 129.3, 130.3, 136.0, 138.0 144.8, 159.0.

**Chemical formula**:	$C_{27}H_{28}N_2O_4$
**Molecular weight**:	444.53 g mol^{-1}
**Yield:**	1.16 g, 2.61 mmol, 66 %
**HRMS** (ESI, pos.,:	Calc. $[M+H]^+$: 445.2122
ACN/water, 1/1, 0.1% TFA)	Found: *m/z* = 303.1365 $[DMT]^+$, 445.2109 $[M+H]^+$

1.3 Synthesis of the phosphoramidite (4) (*R/S*)

Air-sensitive phosphoramidites were handled in a *GS-systems Glovebox* under dry argon atmosphere. Solvents were purified and dried over absorbent-filled columns on a *GS-Systems solvent purification system* (SPS). A solution of (**3**) (0.77 g, 1.73 mmol, 1 equiv.) and DIPEA (1.8 mL, 10.4 mmol, 6 equiv.) in dry CH_2Cl_2 was degassed three times. To the degassed reaction mixture 2-Cyanoethyl *N,N*-diisopropylchlorophosphoramidite (0.46 mL, 2.08 mmol, 1.2 equiv.) was dropwise added and stirred for 90 minutes. After completion of the reaction, the solvent was removed under reduced pressure and the crude product was used for DNA solid-phase synthesis assuming a yield of 80 %.

Chemical formula: $C_{36}H_{45}N_4O_5P$

Molecular weight: 644.75 g mol^{-1}

^{31}P-NMR (242 MHz): σ (/ppm) = 149.0, 149.8.

2 DNA synthesis and purification

2.1 Oligonucleotides

Modified Oligonucleotides were synthesized by solid phase synthesis using the standard protocol with extended coupling times for the imidazole modification on a *K&A Laborgeraete H-8 DNA synthesizer*.[10] Cartridges (*Biosearch Technologies* or *Link Technologies*) for solid phase synthesis were self-packed with CPG (1000 Å, 33 µMol g^{-1}) purchased from *Sigma Aldrich*. All solvents were bought in anhydrous quality. Standard 4,4'-dimethoxytrityl (DMT) and cyanoethyl (CE) protected phosphoramidites (DMTdT-CEP, DMT-dG(iBu)-CEP, DMT-dA(Bz)-CEP) were purchased from *Sigma Aldrich* and were dissolved for DNA synthesis in ACN (0.1 M).

2.2 Reversed phase purification

DNA samples were purified with the DMT-group still attached as a purification tag using reversed phase *Agilent Zorbax 300 SB-C18 9.4 x 250 mm* columns on an *Agilent Technologies 1260 Infinity I* or *II* HPLC system. After purification of the oligonucleotides, the DMT-group was removed with 2 % aqueous TFA on *SepPak C18* cartridges, washed with water and eluted with a water/acetonitrile mixture. The volume was reduced using *H.Saur S-Concentrator BA-VC-300H* vacuum centrifuges to 300 µL and samples were stored at – 20 °C until use. Concentrations were individually determined using a *Thermo Scientific Nanodrop One* instrument under consideration of the molar extinction coefficient of the imidazole-functionalized modification.

3 Thermal denaturation studies

3.1 UV-Vis spectroscopy

All UV-Vis based measurements were carried out on *Jasco V-650* and *Jasco V-750* absorption spectrometers in quartz glass cuvettes. Samples were prepared with 3.75 µM ssDNA in 100 mM NaCl and 10 mM LiCaCo pH 7.2 and, if present, 0.9375 µM transition metal cations. To form the G-quadruplexes, solutions were heated to 85 °C and cooled down to 4 °C at 0.5 °C min^{-1}. Samples were then frozen at –20 °C for one hour to ensure full G-quadruplex formation.

Thermal difference spectra (TDS) were recorded from 220 to 350 nm by subtracting the low temperature spectrum (4 °C) from the high temperature spectrum (85 °C). The average absorption from 340 to 350 nm was then set to 0.

Thermal denaturation experiments were carried out to determine the thermal stability expressed in the melting temperature $T_{1/2}$. Therefore, the change in absorbance, as the temperature was increased with 0.5 °C min^{-1}, was followed at 295 nm indicative for G-quadruplex denaturation and at 350 nm as control. The spectrometer bandwidth was set to 2 nm. To control the temperature, a cuvette containing water was equipped with a temperature probe connected to the spectrometer. Water evaporation from the samples was avoided by addition of small amounts of silicon oil. To ensure equal heating rates between different experiments, always five samples were measured at a time. The measured absorbance was plotted against the

temperature after the absorbance at 350 nm was subtracted to remove background absorption. The observation of spikes at around 5 °C in some melting profiles was caused by an onset of water vapor condensation on the cuvettes. Spectra were normalized to fraction folded values between 1 and 0 corresponding to fully folded or unfolded G-quadruplexes, respectively.

3.2 G_5L^R

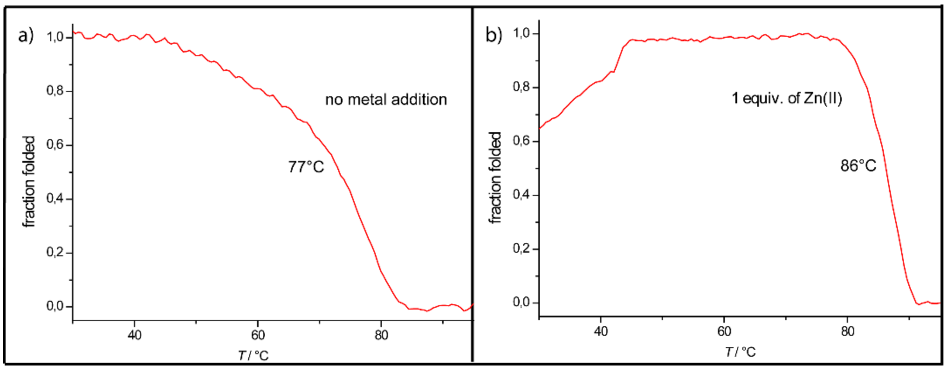

Figure 1. Fraction folded thermal denaturation experiments monitored at λ = 295 nm for the G-quadruplex $G_5\mathbf{L}^R$ a) in absence of transition metals and b) after addition of 1 equiv. Zn(II). Conditions: 3.75 µM DNA, 100 mM NaCl, 10 mM LiCaco pH 7.2 and, if present, 0.9375 µM of the respective metal.

3.3 G_5L^S

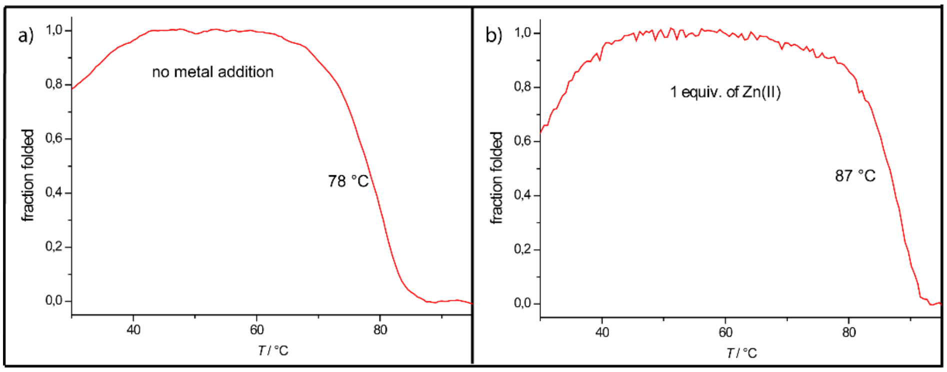

Figure 2. Fraction folded, thermal denaturation experiments monitored at λ = 295 nm for the G-quadruplex $G_5\mathbf{L}^S$ a) in absence of transition metals and b) after addition of 1 equiv. Zn(II). Conditions: 3.75 µM DNA, 100 mM NaCl, 10 mM LiCaco pH 7.2 and, if present, 0.9375 µM of the respective metal.

3.4 G_4L^R

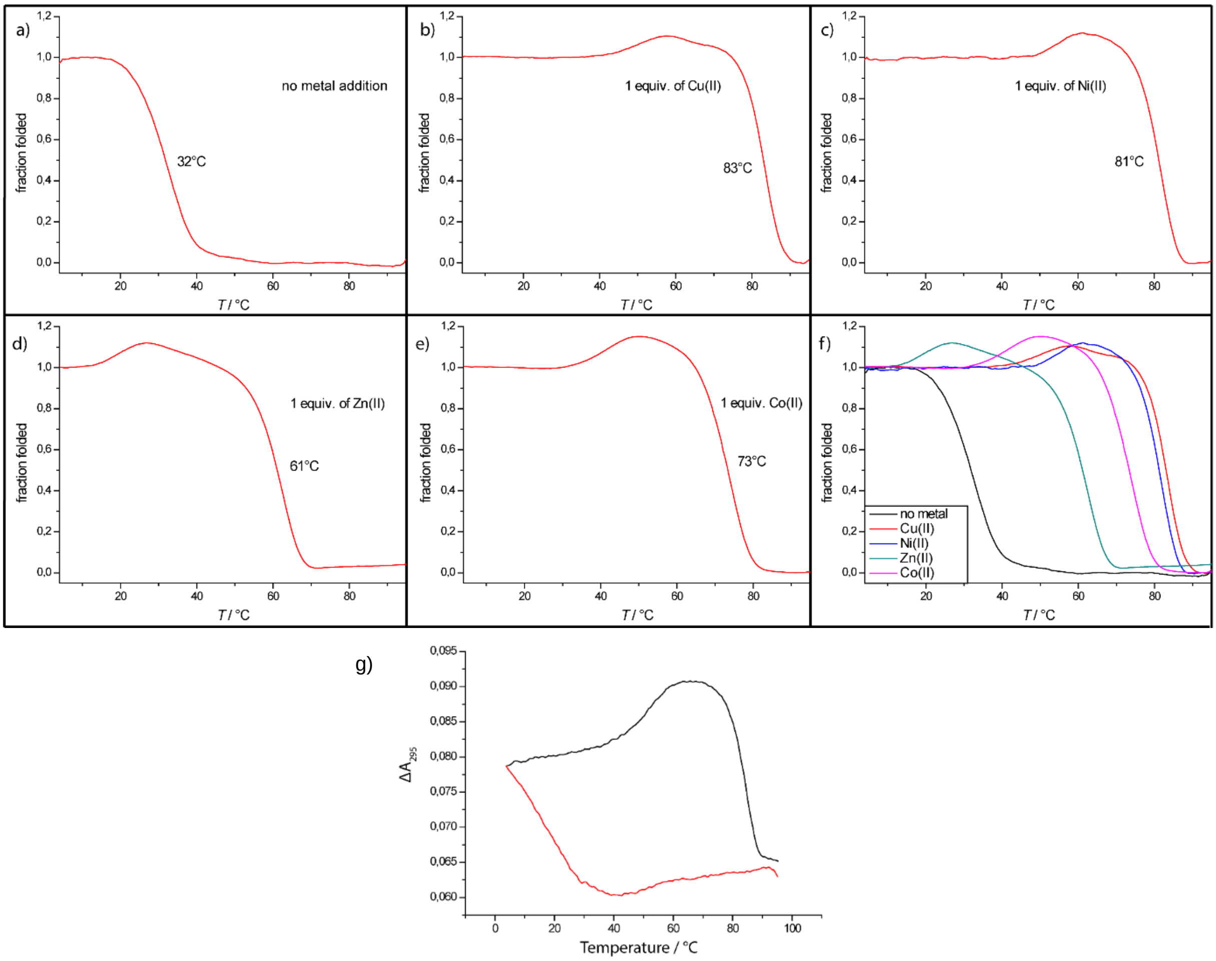

Figure 3. Fraction folded, thermal denaturation experiments monitored at λ = 295 nm for the G-quadruplex $G_4\mathbf{L}^R$ a) in absence of transition metals, after addition of 1 equiv. b) Cu(II), c). Ni(II), d) Zn(II), e) Co(II) and f) superimposition of a-e). Conditions: 3.75 µM DNA, 100 mM NaCl, 10 mM LiCaco pH 7.2 and, if present, 0.9375 µM of the respective metal. g) Denaturing (black) and renaturing (red) profiles of G_4L^R (not normalized). Conditions: 8 µM DNA, 100 mM NaCl, 10 mM LiCaco pH 7.2 and 2 µM Cu(II), heating/cooling rate 0.5 °C/min.

3.5 G_4L^S

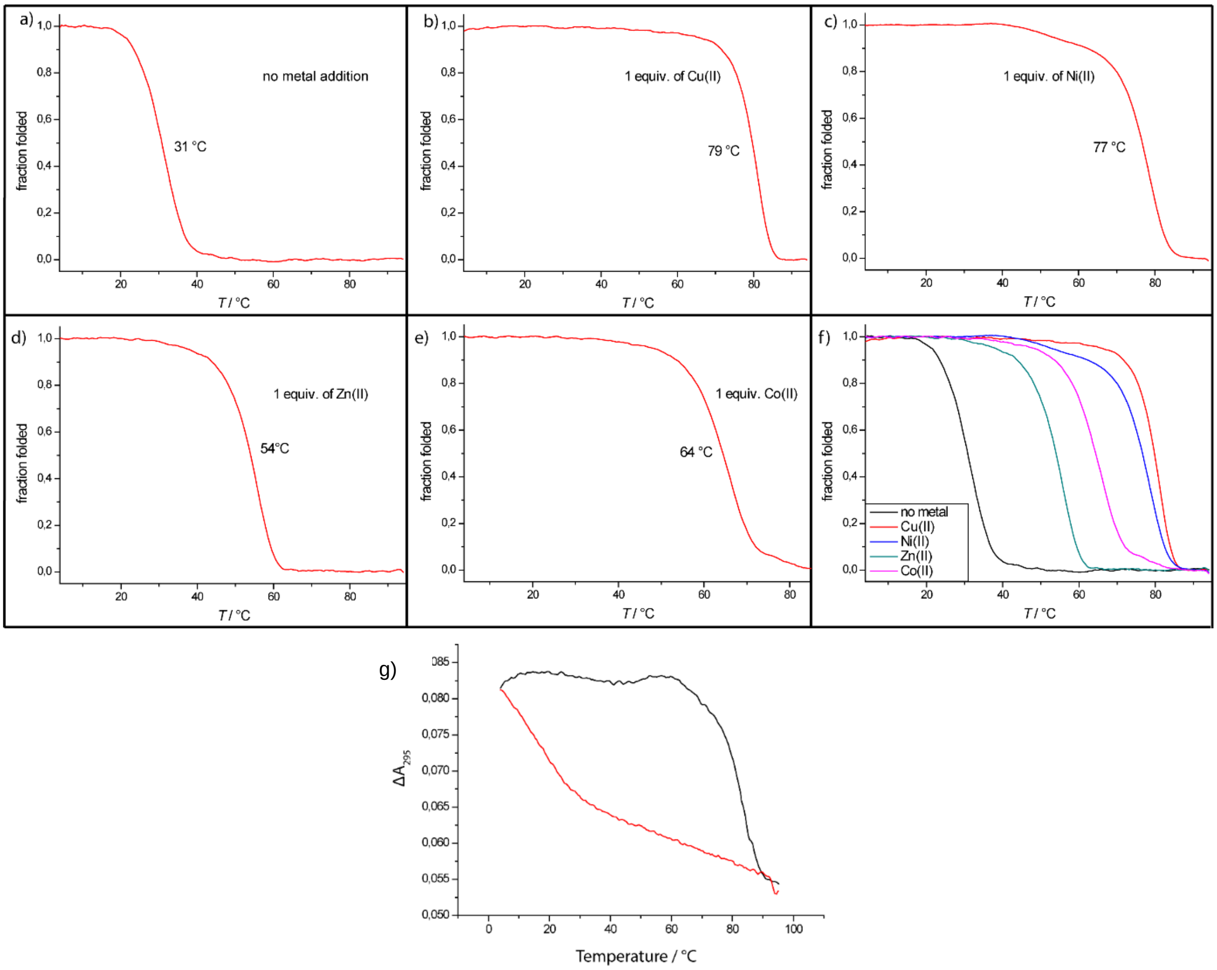

Figure 4. Fraction folded, thermal denaturation experiments monitored at λ = 295 nm for the G-quadruplex $G_4\mathbf{L}^S$ a) in absence of transition metals, after addition of 1 equiv. b) Cu(II), c) Ni(II), d) Zn(II), e) Co(II) and f) superimposition of a-e). Conditions: 3.75 μM DNA, 100 mM NaCl, 10 mM LiCaco pH 7.2 and, if present, 1 equiv. of the respective metal. g) Denaturing (black) and renaturing (red) profiles of G_4L^S (not normalized). Conditions: 8 μM DNA, 100 mM NaCl, 10 mM LiCaco pH 7.2 and 2 μM Cu(II), heating/cooling rate 0.5 °C/min.

3.6 G3L^R and G3L^S in presence of 1 equiv. Cu(II)

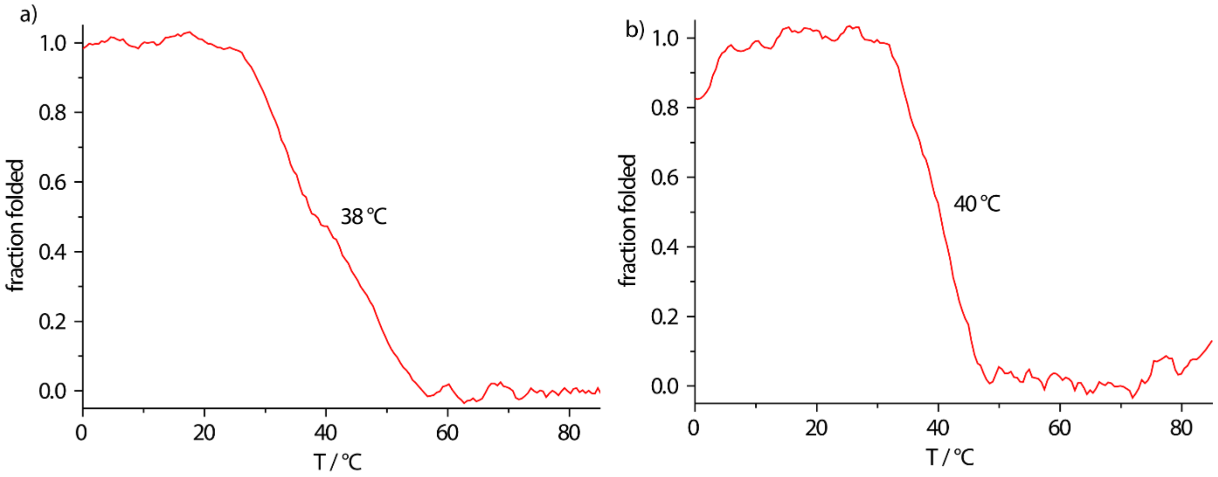

Figure 5. Fraction folded, thermal denaturation experiments monitored at λ = 295 nm for the G-quadruplex a) G3**L**^{R} and b) G3**L**^{S} in presence of 1 equiv. Cu(II). Conditions: 3.75 μM DNA, 100 mM NaCl, 10 mM LiCaco pH 7.2, if present, 0.9375 μM of the respective metal.

3.7 G3L^R and G3L^S in presence of 1 equiv. Cu(II) and 10 equiv. EDTA

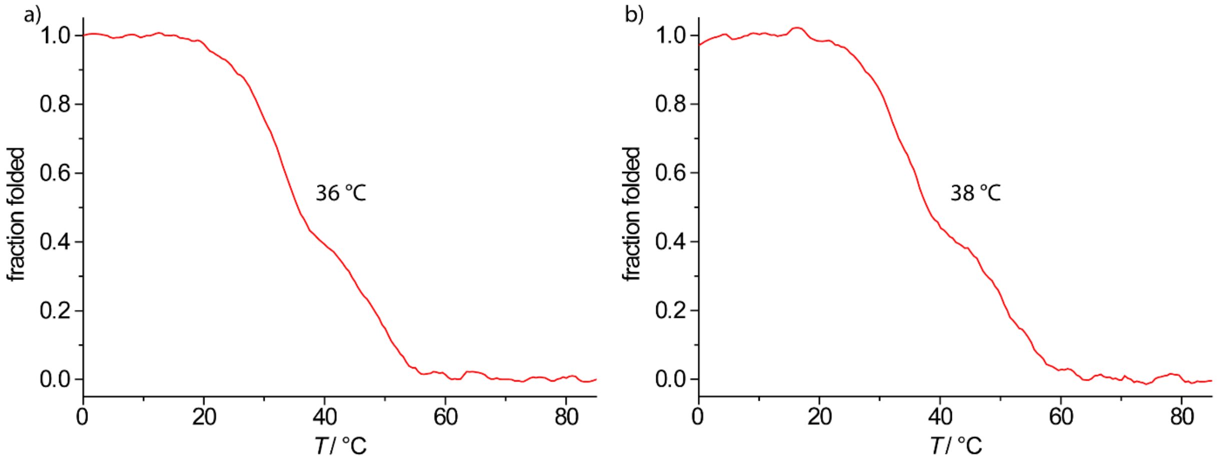

Figure 6. Fraction folded, thermal denaturation experiments monitored at λ = 295 nm for the G-quadruplex a) G3**L**^{R} and b) G3**L**^{S} in presence of 1 equiv. Cu(II) and 10 equiv. EDTA. The G-quadruplex solutions were formed in presence of Cu(II). EDTA was added prior to the thermal denaturation experiment. Conditions: 3.75 μM DNA, 100 mM NaCl, 10 mM LiCaco pH 7.2, 10 μM EDTA and0.9375 μM Cu(II).

3.8 $G_4\mathbf{L}^S$ stability screen

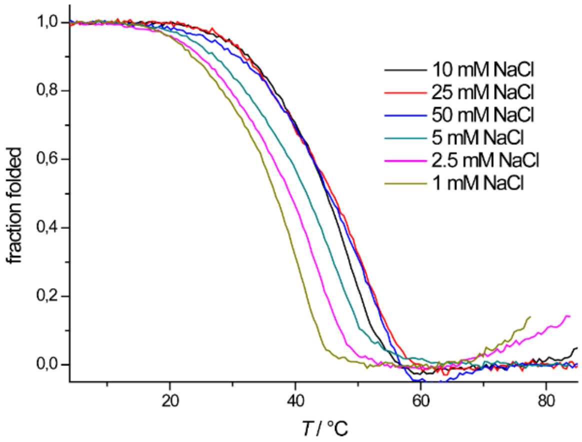

Figure 7. Melting curves of $G_4\mathbf{L}^S$ in presence of Hemin with varying concentrations of NaCl. Conditions: 3.75 µM DNA, 1-50 mM NaCl, 10 mM LiCaco pH 7.2.

3.9 $G_4\mathbf{L}^S$ in presence of 10 equiv. EDTA

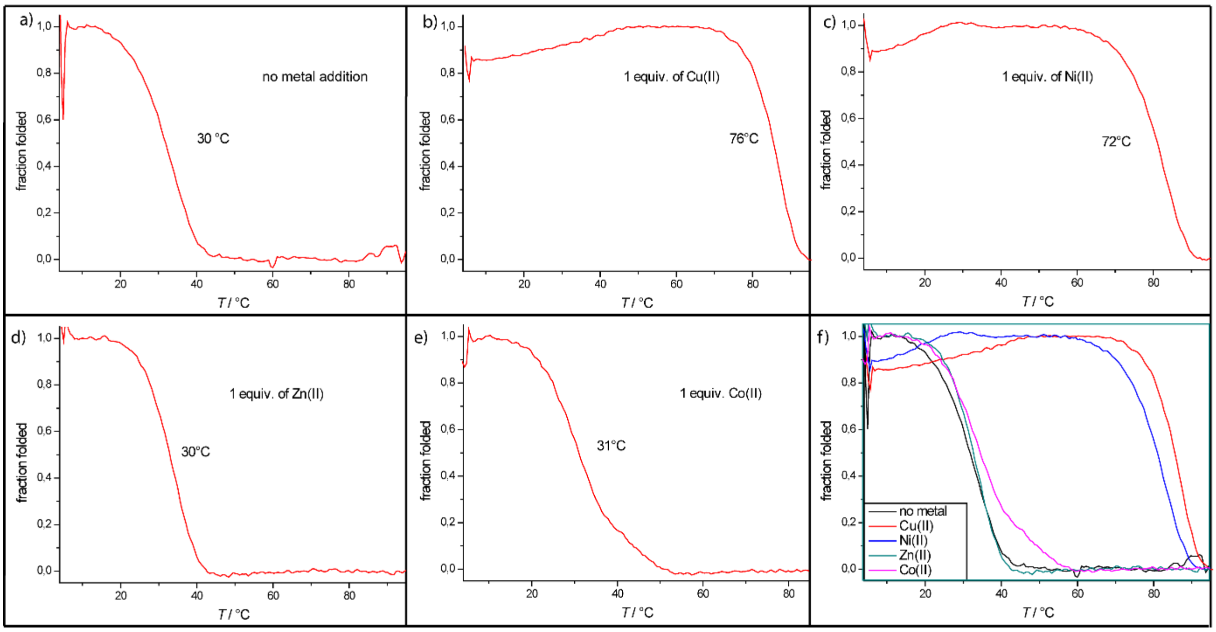

Figure 8. Fraction folded, thermal denaturation experiments monitored at λ = 295 nm for the G-quadruplex $G_4\mathbf{L}^S$ in presence of 10 equiv. EDTA a) in absence of transition metals, after addition of 1 equiv. b) Cu(II), c). Ni(II), d) Zn(II), e) Co(II) and f) superimposition of a-e). Conditions: 3.75 µM DNA, 100 mM NaCl, 10 mM LiCaco pH 7.2, 10 µM EDTA and, if present, 1 equiv. of the respective metal.

3.10 G4L^{R} in presence of 10 equiv. EDTA

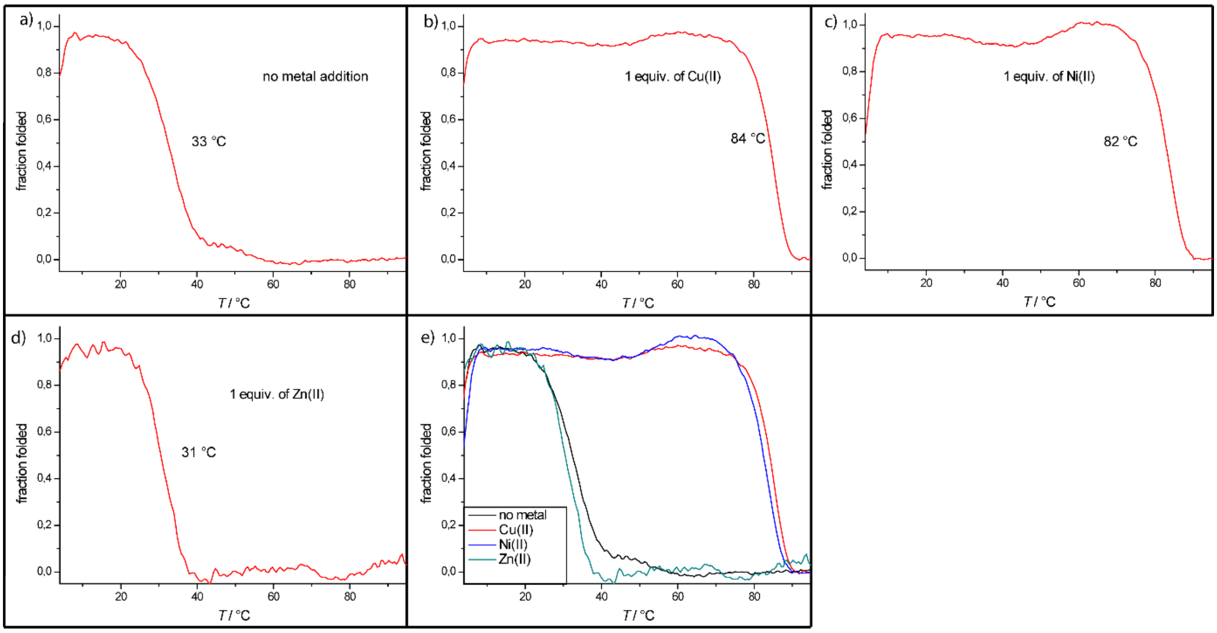

Figure 9. Fraction folded, thermal denaturation experiments monitored at λ = 295 nm for the G-quadruplex G$_4$**L**^{R} in presence of 10 equiv. EDTA a) in absence of transition metals, after addition of 1 equiv. b) Cu(II), c). Ni(II), d) Zn(II), e) Co(II) and f) superimposition of a-e). Conditions: 3.75 μM DNA, 100 mM NaCl, 10 mM LiCaco pH 7.2, 10 μM EDTA and, if present, 1 equiv. of the respective metal.

4 Thermal difference spectra (TDS)

4.1 G$_5$L^{RS}

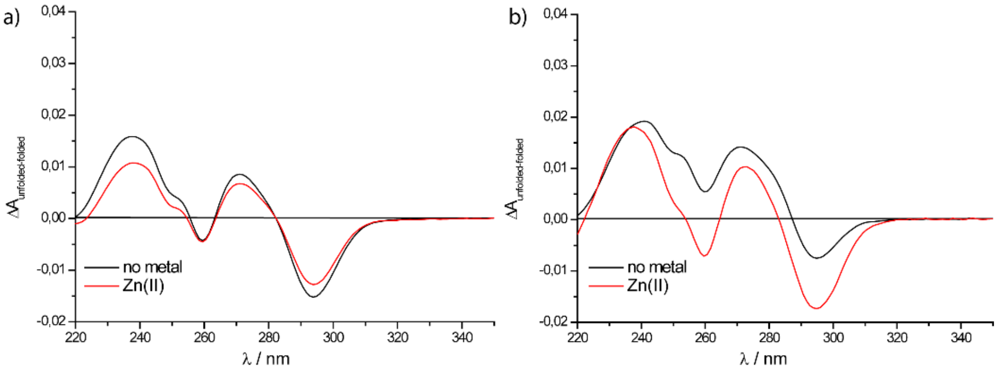

Figure 10. TDS of G$_5$**L**^{RS} recorded from 220 to 350 nm. a) G$_5$L^{R} and b) G$_5$L^{S}. Conditions: 3.75 μM DNA, 100 mM NaCl, 10 mM LiCaco pH 7.3 and, if present, 3.75 μM of the respective metal.

4.2 G$_4$L^R

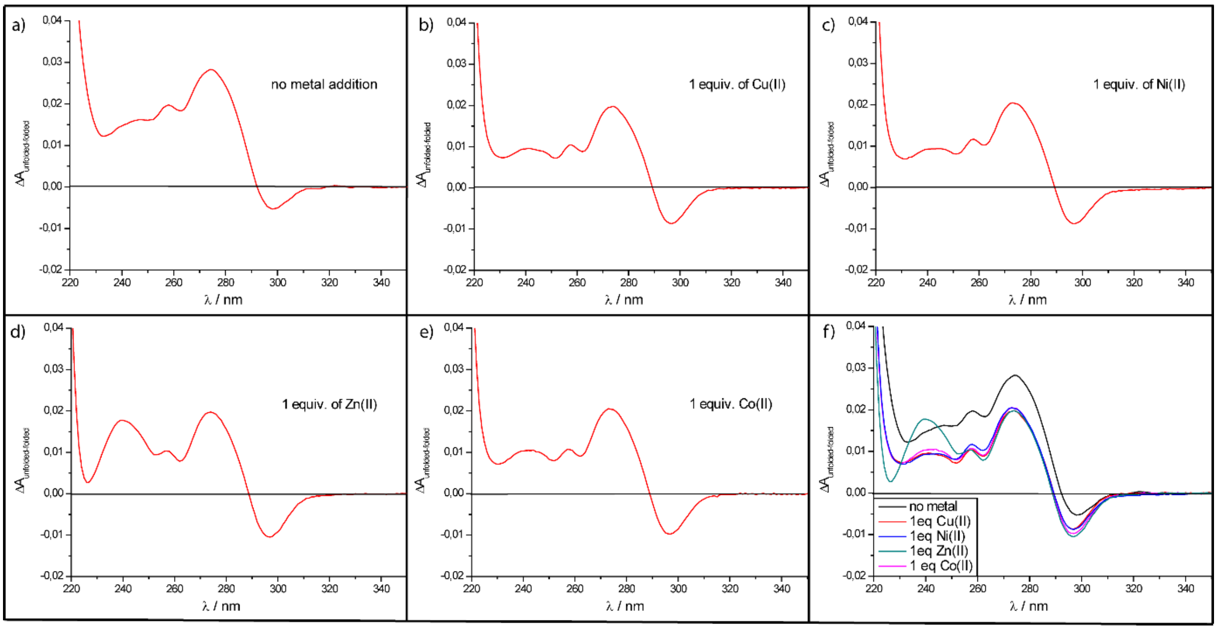

Figure 11. TDS of G$_4$L^R recorded from 220 to 350 nm. a) in absence of transition metals, after addition of 1 equiv. b) Cu(II), c). Ni(II), d) Zn(II), e) Co(II) and f) superimposition of a-e). Conditions: 3.75 µM DNA, 100 mM NaCl, 10 mM LiCaco pH 7.3 and if present 3.75 µM of the respective metal.

4.3 G$_4$L^S

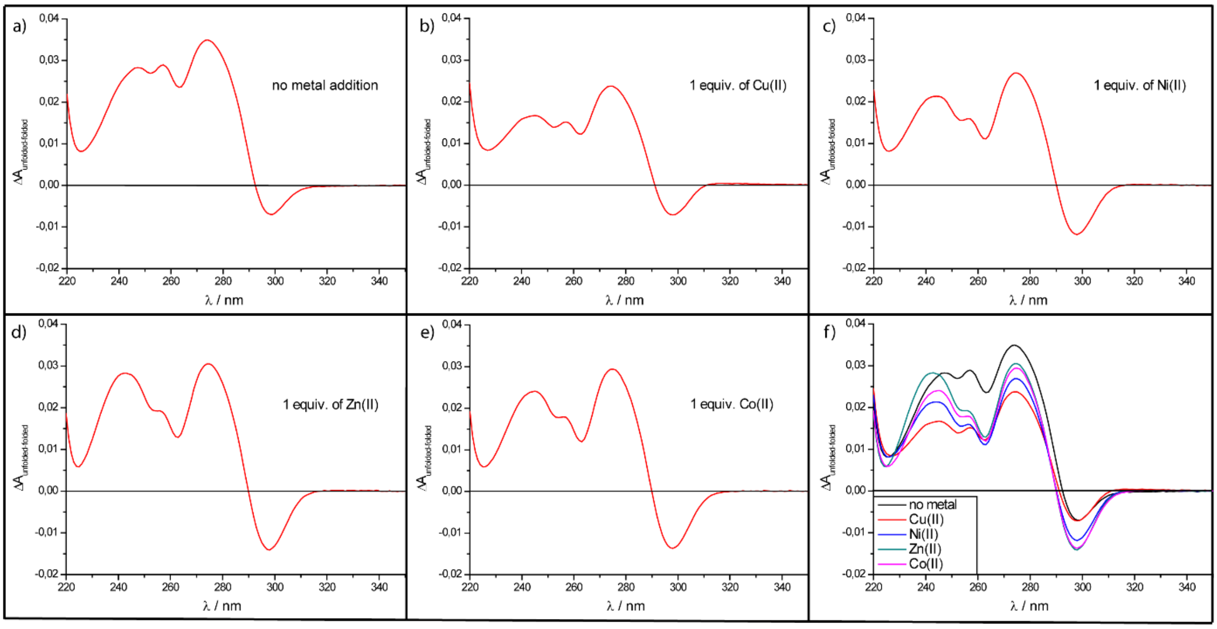

Figure 12. TDS of G$_4$L^S recorded from 220 to 350 nm. a) in absence of transition metals, after addition of 1 equiv. b) Cu(II), c). Ni(II), d) Zn(II), e) Co(II) and f) superimposition of a-e). Conditions: 3.75 µM DNA, 100 mM NaCl, 10 mM LiCaco pH 7.3 and, if present, 3.75 µM of the respective metal.

5 Circular dichroism measurements

5.1 CD spectroscopy

CD measurements were carried out on a *Chirascan qCD* spectrometer in black quartz-glass cuvettes. G-quadruplex samples were prepared as previously described (SI 3.1). The temperature was controlled using a Quantum Northwest temperature control attached to a sample probe. Spectra were recorded at 7°C from 205 to 350 nm (120 nm min^{-1}) with a 1 nm interval and 0.5 nm bandwidth three times and averaged using the instrument-specific software. The averaged spectra were smoothed (adjacent averaging) with a factor of 5 and the smoothed background (sample containing only buffer, smooth factor 10) was subtracted. The background was recorded from 205 to 350 nm with the same settings as for the samples.

5.2 G_5L^{RS}

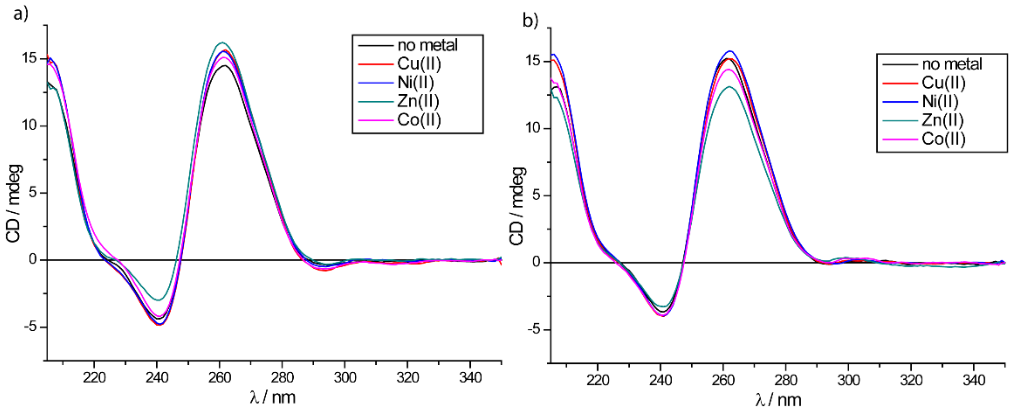

Figure 13. CD spectra of the folded G-quadruplex a) $G_5\mathbf{L}^{R}$ and b) $G_5\mathbf{L}^{S}$ at 4 °C displayed in milli-degree. Conditions: 3.75 μM DNA, 100 mM NaCl, 10 mM LiCaco pH 7.3 and, if present, 3.75 μM of the respective metal.

5.3 G_4L^R

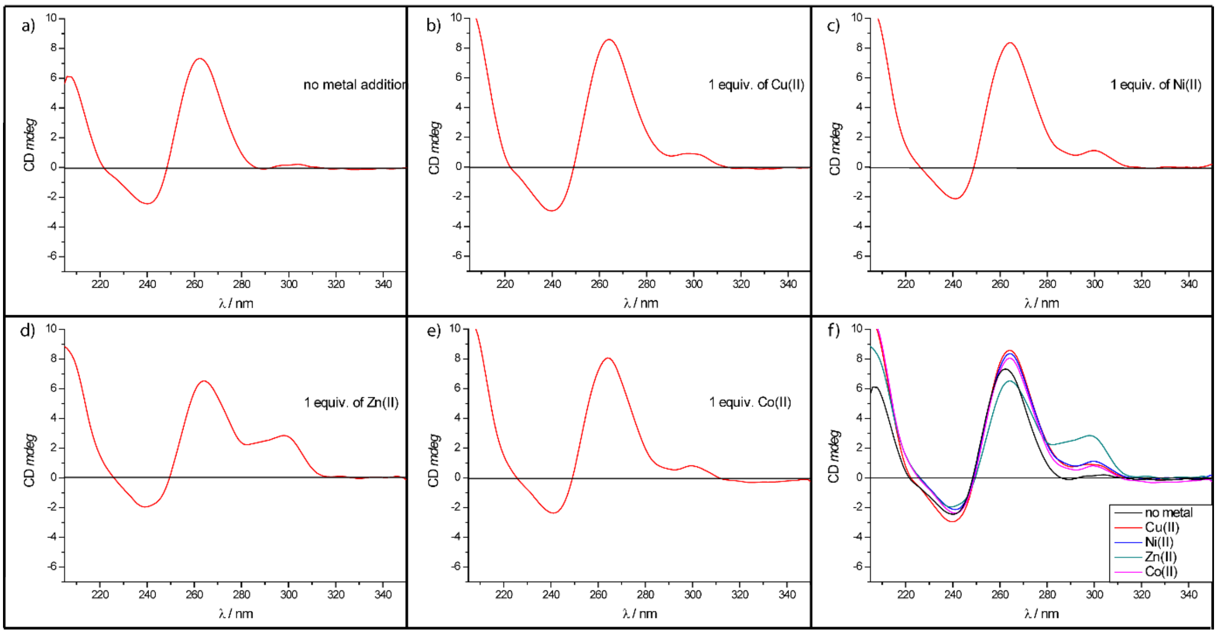

Figure 14. CD spectra of the folded G-quadruplex $G_4\mathbf{L}^R$ at 4 °C displayed in milli-degree. a) in absence of transition metals, after addition of 1 equiv. b) Cu(II), c). Ni(II), d) Zn(II), e) Co(II) and f) superimposition of a-e). Conditions: 3.75 µM DNA, 100 mM NaCl, 10 mM LiCaco pH 7.3 and, if present, 3.75 µM of the respective metal.

5.4 G_4L^S

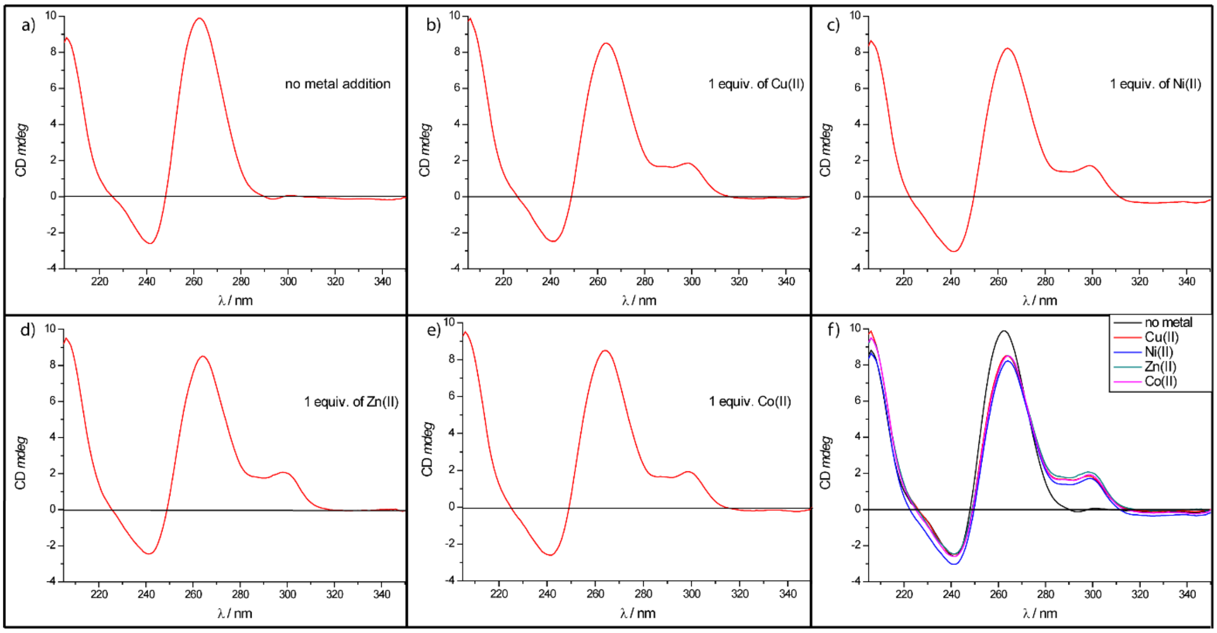

Figure 15. CD spectra of the folded G-quadruplex $G_4\mathbf{L}^S$ at 4 °C displayed in milli-degree. a) in absence of transition metals, after addition of 1 equiv. b) Cu(II), c). Ni(II), d) Zn(II), e) Co(II) and f) superimposition of a-e). Conditions: 3.75 µM DNA, 100 mM NaCl, 10 mM LiCaco pH 7.3 and, if present, 3.75 µM of the respective metal.

5.5 G_3L^R and G_3L^S

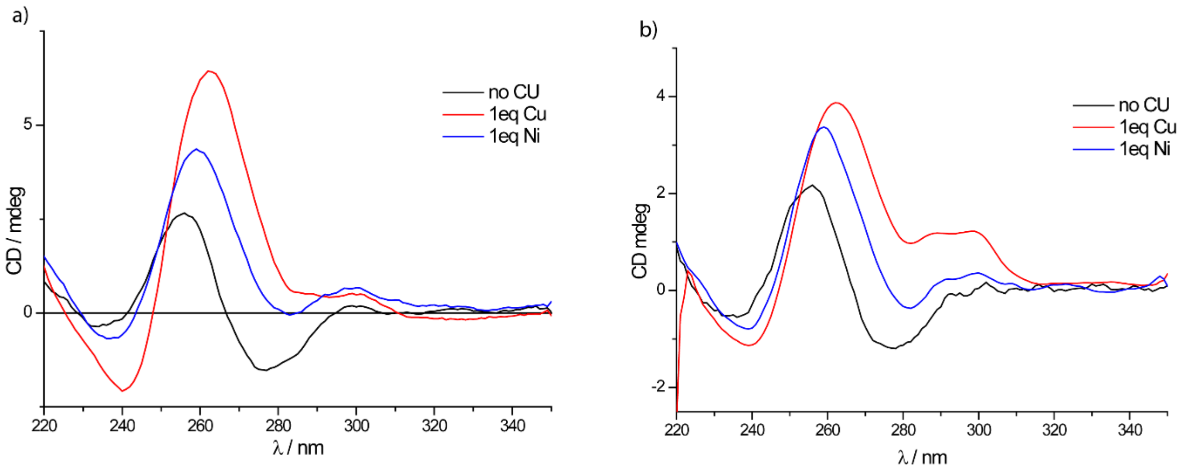

Figure 16. CD spectra of a) G_3L^R and b) G_3L^S at 7 °C. Conditions: 3.75 µM DNA, 100 mM NaCl, 10 mM LiCaCo pH 7.3 and, if present, 1 equiv. of the respective metal.

5.6 Time-resolved CD-spectroscopy of G_3L^R and G_3L^S after EDTA addition

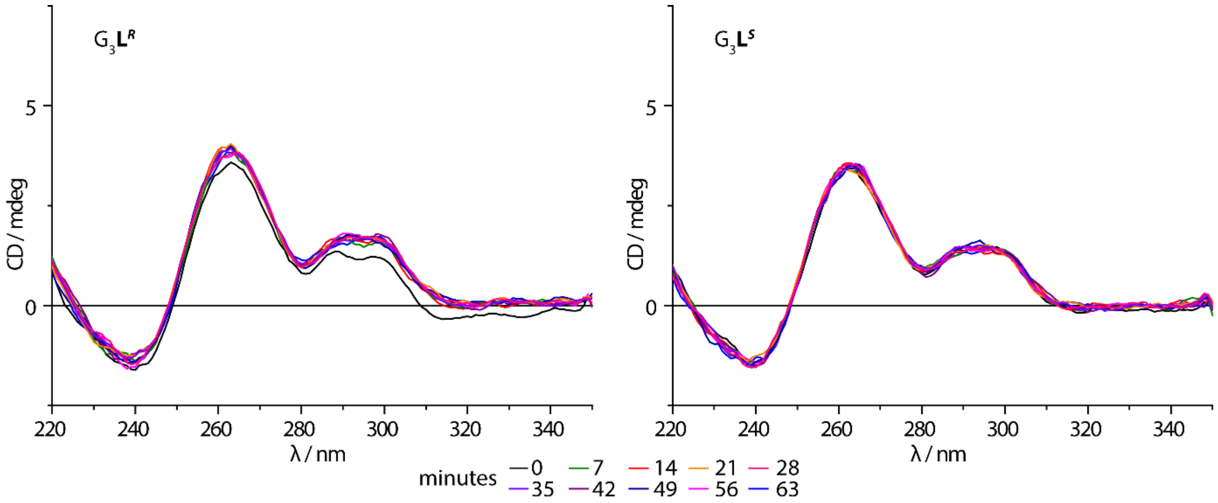

Figure 17. CD spectra of G_3L^R and G_3L^S at 7 °C. Conditions: 3.75 µM DNA, 100 mM NaCl, 10 mM LiCaCo pH 7.3 and 1 equiv. Cu(II). The measurement was started after addition of 10 equiv. EDTA.

6 MD simulations

Simulations were performed using GROMACS 2016.1.[3-9] The AMBER force field *ff99bsc1* was modified with new force field parameters regarding the imidazole ligand according to a previously published protocol.[10-14] Missing forcefield parameters for the imidazole ligand were based on analogy to existing force field parameters. For both enantiomers, the same set of parameters was used. Parameters regarding the Cu(II) and Zn(II) complex were derived from literature using VFFDT (Visual Force Field Derivation Toolkit) with a Gaussian '09 geometry optimized structure (B3LYP/6-311+G(d,p)).[10,15-17,28] To obtain a square-planar coordination environment for Cu(II), adjacent ligands were named differently, to avoid ambiguity in the parameterization. Improper dihedrals were used to maintain coplanarity between the imidazole plane and the imidazole – metal plane. The respective parameters for Cu(II) and Zn(II) were estimated based on literature values(table 2).[17,28]

As starting structure, the crystal structure of a tetramolecular parallel stranded G-quadruplex (pdb entry: 2O4F) was used and modified.[18] Obtained starting structures where solvated in a rhombic bounding box with TIP3P water. Negative charges on the phosphates were neutralized with Na^+ and the concentration of NaCl was set to 100 mM. Prior to the MD simulation,

the starting structure was subjected to three rounds of energy minimization and equilibrated at 298 K and 1 bar. The final MD was simulated for 50 ns with 0.5 fs timesteps in an NPT ensemble. For details see our previous publication.[10]

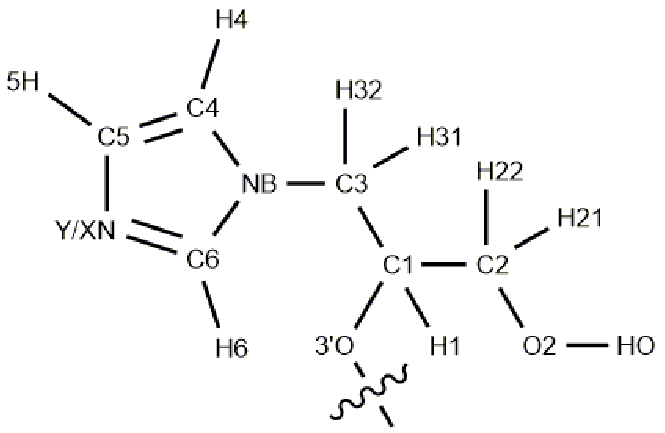

Figure 18. Atom names of the parameterized imidazole ligand L.

6.1 Partial charges (RESP)

Partial point charges were derived by RESP charge fitting. Therefore the imidazole ligands and the imidazole Cu(II) complex were fragmented according to the original capping scheme, structure optimized (B3LYP/6-311+G(d,p)) and submitted to the REDServer-Development (table 1). Inter-molecular and intra-molecular charge constraints were used to maintain the correct total charge of the ligand. In all cases, the constrained charges were similar to the unconstrained values.[19-22]

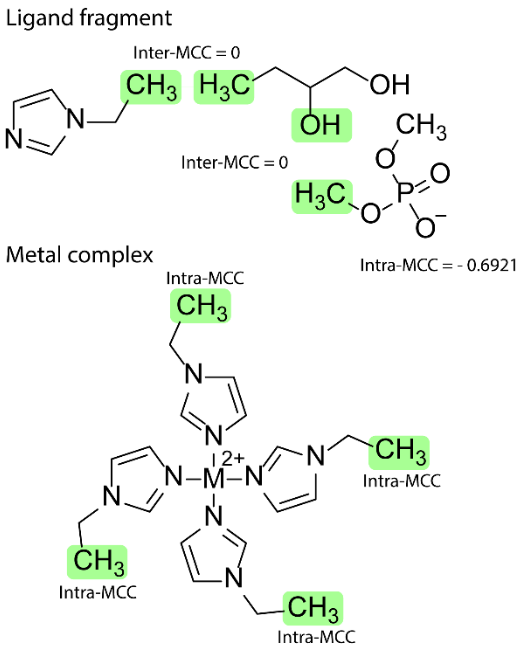

Figure 19: Applied RESP-Capping Scheme for the imidazole Ligand and the respective imidazole-metal complexes.

Supporting Information

Table 1: Calculated partial charges for the imidazole Ligand L and in complex with Cu(II) and Zn(II)

atom name	5' ligand	Ligand free	Ligand coord. to Cu	Ligand coord. to Zn
O3'	OS	-0,6534	-0,6534	-0,6534
C1	CI	0.6009	0.6009	0.6009
H1	H1	-1.248	-1.248	-1.248
C2	CT	0.1565	0.1565	0.1565
H21	H1	0.0065	0.0065	0.0065
H22	H1	0.0065	0.0065	0.0065
O2	OH	-0.6678	-0.6678	-0.6678
HO	HO	0.4261	0.4261	0.4261
C3	CT	-0.0297	-0.01765	-0.0782
H31	H1	0.0561	0.1097	0.1062
H32	H1	0.0561	0.1097	0.1062
N1	NB	0.0452	0.0329	0.0074
C4	CV	-0.3696	-0.2091	-0.1921
H4	H4	0.2204	0.2321	0.2553
C5	CW	0.0782	-0.1819	-0.2194
H5	H4	0.1377	0.2014	0.2054
NX/Y	NX/Y	-0.5352	-0.1370	0.0143
C6	CR	0.1448	0.06470	-0.0114
H6	H5	0.1366	0.1443	0.1916
Zn	Zn			0.2252
CU	CU		0.3699	

Table 2: Forcefield parameters regarding the imidazole ligand L and the respective metal complexes with Cu and Zn

Bond				bond length b_0 [nm]	force constant k_b [kJ mol^{-1} nm^{-2}]	comment
CT	NB	1		0.1475	282001.6	Same as CT N*
CI	CI	1		0.1526	259408	Same as CE CT
CR	NX/Y	1		0.1335	408358.4	Same as CR NB
CV	NB	1		0.1394	343088	Same as CV NB
CV	CW	1		0.1375	428441.6	Same as CC CV
CW	NX/Y	1		0.1394	343088	Same as CV NB
NX/Y	CU	1		0.20219	69965.4	VFFDT
NX/Y	Zn	1		0.209	61587.8	Literature
**angle**				**angle**	**force constant k_ϑ [kJ mol^{-1} nm^{-2}]**	**comment**
OS	CI	CI	1	109.5	418.4	Same as OS CE CT
CI	CI	H1	1	109.5	418.4	Same as CE CT H1
CT	CI	CI	1	109.5	334.72	Same as CT CE CT
CI	CT	NB	1	109.5	418.4	Same as CT CT N*
CI	CI	OH	1	109.5	418.4	Same as CT CI OH
H1	CT	NB	1	109.5	418.4	Same as H1 CT N*
CW	CV	H4	1	120	418.4	Same as CC CV H4
CV	CW	H4	1	120	418.4	Same as CC CV H4
CR	NB	CT	1	128.8	585.76	Same as CK N* CT
CV	NB	CT	1	128.8	585.76	Same as CK N* CT
CW	CV	NB	1	120	585.76	Same as CW CC NB
CR	NX/Y	CW	1	120	585.76	Same as CR NA CW

NX/Y	CW	CV	1		120	585.76	Same as CC CW NA
CW	CV	NB	1		120	585.76	Same as CC CV NB
NB	CR	NX/Y	1		120	585.76	Same as NA CR NA
H4	CW	NX/Y	1		120	418.4	Same as H4 CW NA
H5	CR	NX/Y	1		120	418.4	Same as H5 CR NA
NX/Y	CU	NY/X	1		90	521.81	Literature
NX/Y	CU	NX/Y	1		180	512.5	Literature
CW	NX/Y	CU	1		126.8	890	VFFDT
CR	NX/Y	CU	1		126.9	857.2	VFFDT
CW	NX/Y	Zn	1		126.6	284.2	Literature
CR	NX/Y	Zn	1		126.6	126.6	Literature
NX/Y	Zn	NY/X	1		109.5	500	Literature
NX/Y	Zn	NX/Y	1		109.5	500	Literature
**improper dihedral**					**angle**	**force constant k_φ [kJ mol^{-1} nm^{-2}]**	**comment**
CW	CR	NX	CU	4	180	30	estimated
CW	CR	NX	Zn	4	180	30	estimated
**proper dihedral**					**anlge**	**force constant k_φ [kJ mol^{-1} nm^{-2}]**	**comment**
X	NX/Y	CR	X	9	180	9.7278	Same as X CR NA X
OS	CI	CI	OH	9	0	4.9162	Same as OS CE CI OH
H1	CI	CI	OH	9	0	1.046	Same as H1 CT CI OH
H1	CI	CI	H1	9	0	0.65084	Same as X CT CT X
H1	CI	CI	OS	9	0	1.046	Same as H1 CI CT OS
H1	CI	CI	CT	9	0	0.66944	same as H1 CI CT CE
CT	CI	CI	OH	9	295.63	0.38535	Same as CT CT CI OH
X	CV	CW	X	9	180	6.276	Same as X CC CW X
X	CT	NB	X	9	0	0	Same as X CT N* X
X	CW	NX/Y	X	9	180	9.7278	Same as X CW NA X
X	NX/Y	CR	X	9	180	9.7278	Same as X CW NA X

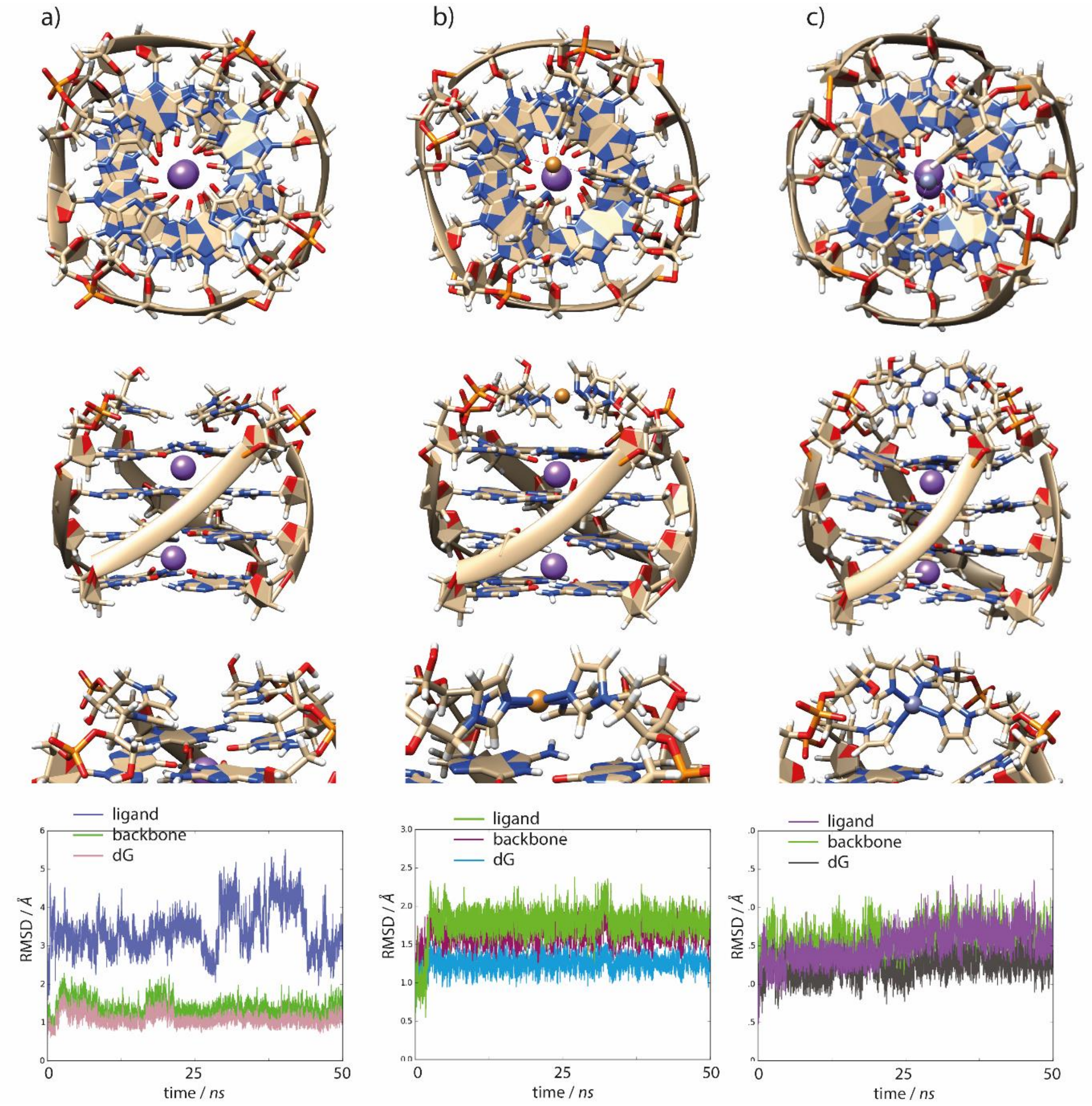

Figure 20. MD simulations of $G_4$$\mathbf{L}^{S}$ in absence (a) and presence of (b) Cu(II) and (c) Zn(II) with the corresponding RMSD graphs. $\mathbf{L}^{S}$ was predominantly found to interact with the 3'-tetrad via π-π-interactions in contrast to $\mathbf{L}^{R}$ which was rather found to interact with the G-quadruplex grooves. This resulted in a reduced ligand dynamic for $\mathbf{L}^{S}$ expressed in smaller RMSD values compared to $\mathbf{L}^{R}$. After metal coordination, the dynamics of L^{S} drastically decreased, expressed in significantly lower RMSD values.

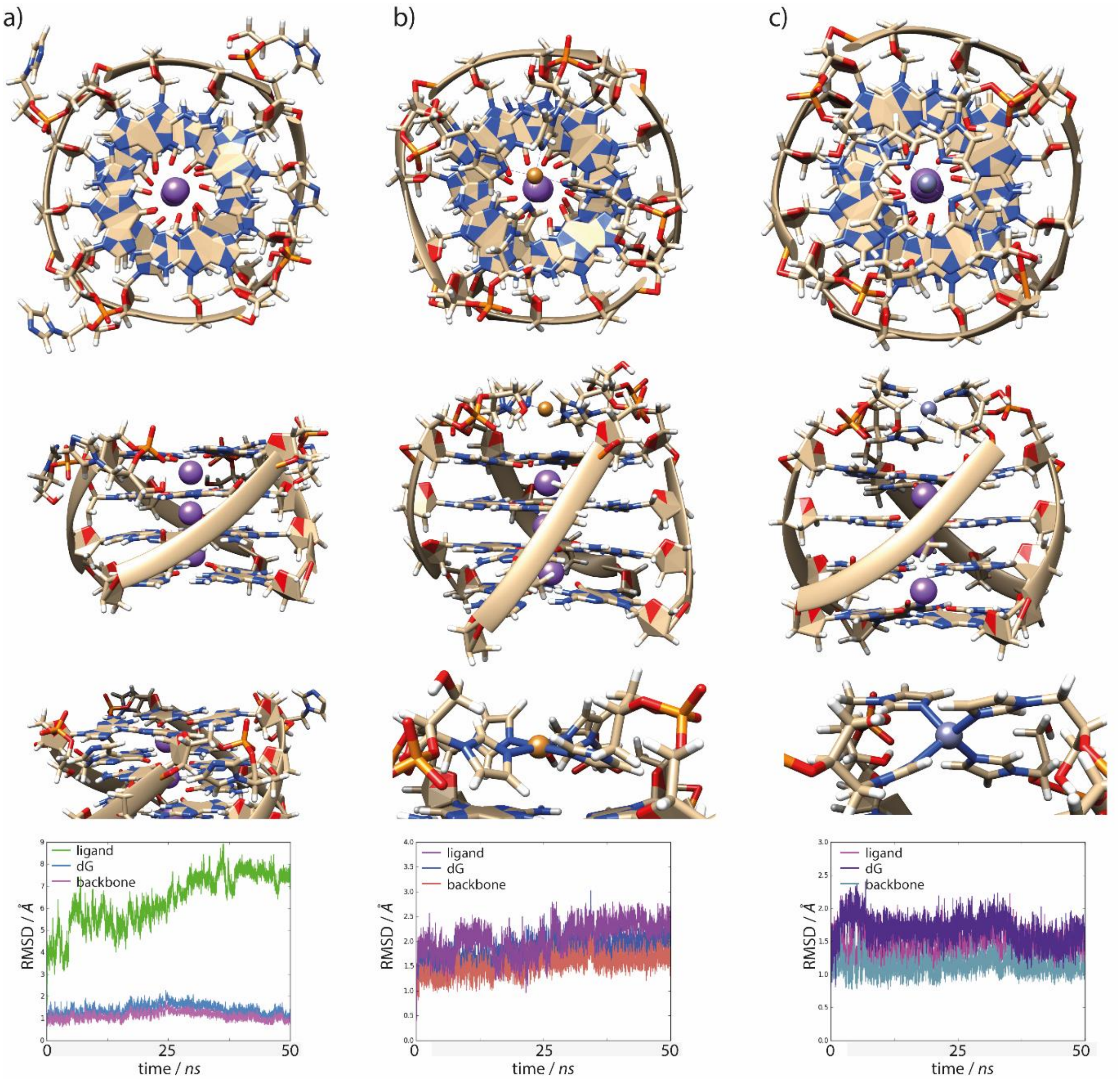

Figure 21. MD simulations of G$_4$**L^R** in absence (a) and presence of (b) Cu(II) and (c) Zn(II) with the corresponding RMSD graphs. **L^R** was predominantly found to interact with the G-quadruplex grooves in contrast to **L^S** which was showing strong π-π-interactions with the 3'-tetrad. This resulted in a higher ligand dynamic for **L^R** expressed in larger RMSD values compared to **L^S**. After metal coordination, the dynamics of **L^R** drastically decreased, expressed in significantly lower RMSD values.

CD-spectroscopy based kinetic studies

G-quadruplex association studies were performed on a *Chirascan qCD* spectrometer in black quartz-glass cuvettes by observing the change of the CD effect at 295 nm at 7 °C for ~ 20 h. Association was followed for every sample at two different concentrations to minimize a potential error of the k_{on} calculation. Sample solutions were prepared at concentrations of 100 mM NaCl, 10 mM LiCaCo pH 7.2 and 8-16 µM single strand. Prior to measurements the solutions were heated to 95 °C for 10 min. The hot sample solutions were transferred to the cuvettes and were temperature equilibrated to 7 °C for 120 s before the measurement was started. The recorded spectra were then converted to the G-quadruplex concentration. k_{on} was calculated according to literature procedures using eq. 1 in which [A$_4$] is the G-quadruplex and [A] the single strand concentration.[23,24]

Equation 1: $$d[A]/d\,t = -4 \cdot d[A_4]/d\,t = -\,k_{on}\,[A]^4$$

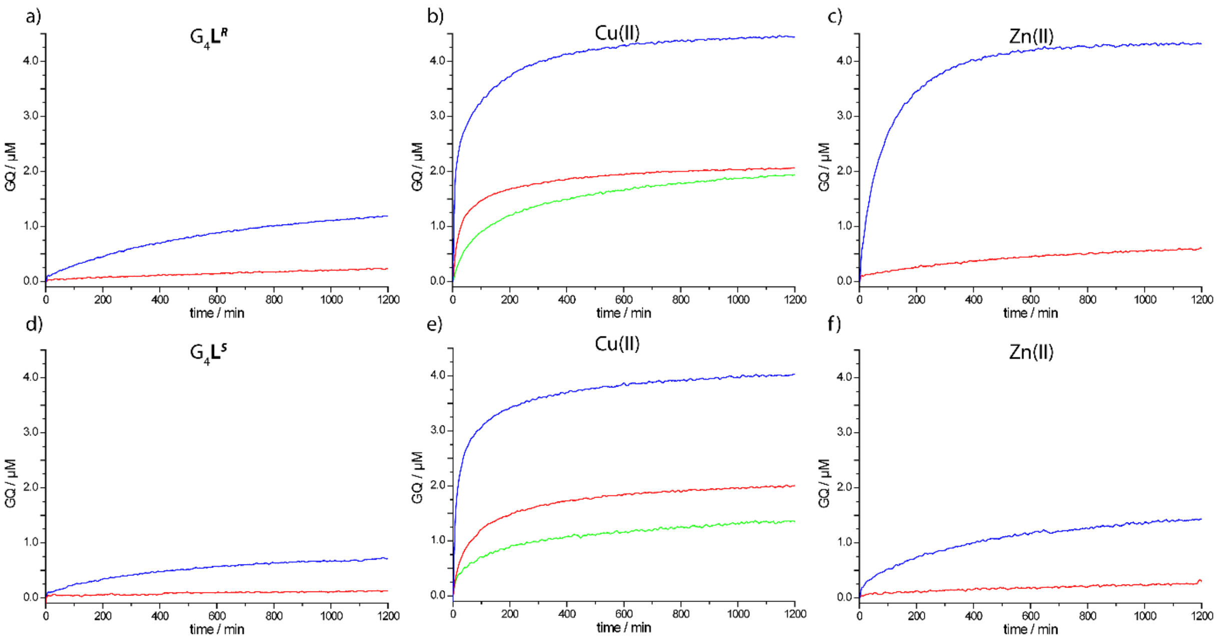

Figure 22. Kinetic GQ association studies of G_4**L**^{RS} at 8 (red) and 16 (blue) µM single strand. If present, 1 equiv. Cu(II) or Zn(II) was added with the exception of the green curves in b) and e) where 10 equiv. Cu(II) were added to 8 µM single strand.

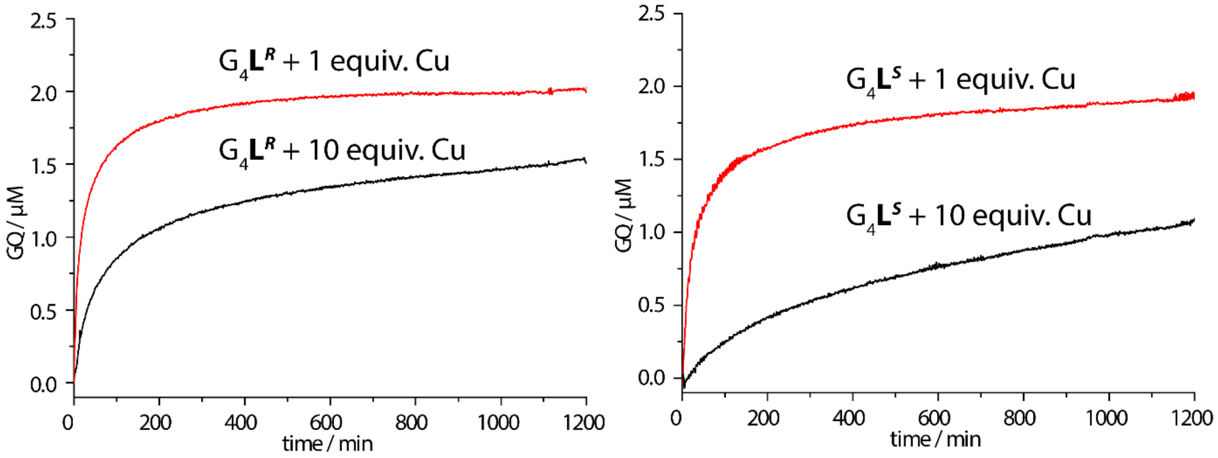

Figure 23. Kinetic GQ association studies of G_4**L**^{RS} at 8 µM single strand in presence of 1 equiv. Cu(II) (red) and 10 equiv. Cu(II) (black).

7 ABTS assay

G-quadruplex stock solutions were prepared at concentrations of 5 µM single strand, 1.25 µM transition metal, 100 mM NaCl and 10 mM HEPES pH 8 by heating to 85 °C for 5 min and slow cooling (0.5 °C min^{-1}) to 4 °C for 16 h. Stock solutions were then frozen at –20 °C for 1h to facilitate full G-quadruplex formation. For the ABTS-assay (2,2'-azino-bis(3-ethylbenzothiazoline-6-sulphonic acid)) the stock solutions were diluted 1/1 with water before Hemin and ABTS were added followed by 10 min incubation at 25 °C. If the assay was performed in presence of EDTA, hemin and EDTA were added simultaneously followed by 1 h of incubation. ABTS was added 10 min prior to H_2O_2 addition. The reaction was started by addition of H_2O_2 and was followed by the change of absorption at 414 nm. The initial reaction rate v_0 was then calculated from the slope of the first 10 s and the extinction coefficient of the ABTS radical (39.000 M^{-1} cm^{-1}).

Final concentrations: 625 nM G-quadruplex, 625 nM transition metal, 50 mM NaCl and 5 mM HEPES pH 8, 500 nm Hemin, 2 mM ABTS, 0.5 mM H_2O_2.

7.1 ABTS assay with G_4L^S

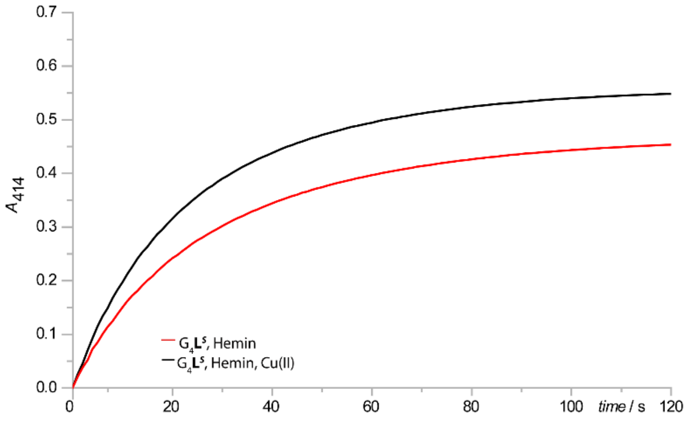

Figure 24. ABTS assay with G_4L^S.

7.2 ABTS assay with G_3L^S.

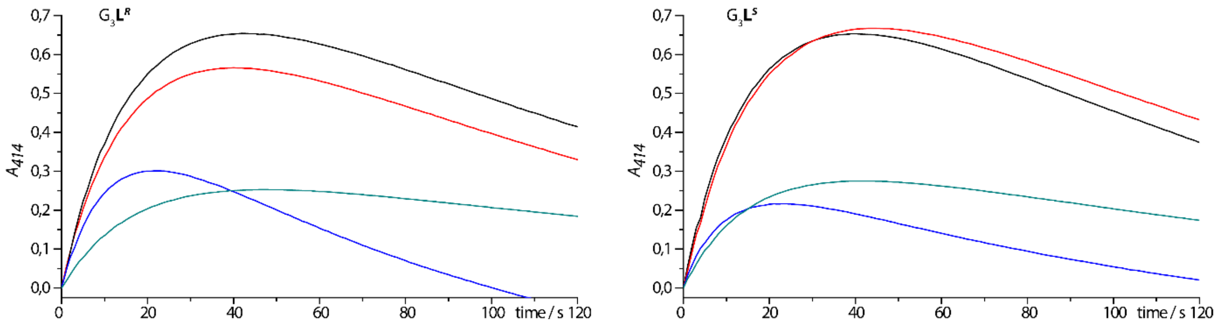

Figure 25. ABTS assay with G_3L^R and G_3L^S in presence of 1 equiv. Cu(II) (black); 1 equiv. Cu(II), Fe(II), Mn(II), Zn(II) and Co(II) (red); 1 equiv. Ni(II) (blue); 1 equiv. Cu(II) and 10 equiv. EDTA (green). EDTA was added after G-quadruplex formation together with Hemin followed by 1 h incubation at 25 °C.

8 Native ESI mass spectrometry

8.1 Sample preparation

For native ESI-MS experiments KCl instead of NaCl was used ensuring a higher stability of the G-quadruplex and LiCaCo was replaced by volatile TMAA (trimethyl ammonium acetate). Samples were prepared at 100 µM ssDNA, 1 mM KCl and 100 mM TMAA pH 6.8 and, if used, 25 µM $Cu(II)SO_4$. To form G-quadruplexes, solutions were heated to 85 °C and cooled down to 4 °C at 0.5 °C min^{-1}. Prior to measurements, samples were diluted 1:1 with acetonitrile to final concentrations of 12.5 µM G-quadruplex, 0.5 mM KCl and 50 mM TMAA pH 6.8.

8.2 Trapped Ion Mobility ESI Mass Spectrometry

Ion mobility measurements were performed on a Bruker timsTOF instrument combining a trapped ion mobility (TIMS) with a time-of-flight (TOF) mass spectrometer in one instrument.

In contrast to the conventional drift tube method to determine mobility data, where ions are carried by an electric field through a stationary drift gas, the TIMS method is based on an electric field ramp to hold ions in place against a carrier gas

pushing them in the direction of the analyzer. Consequently, larger sized ions that experience more carrier gas impacts leave the TIMS units first and smaller ions elute later. This method offers a much higher mobility resolution despite a smaller device size.

Measurement: After the generation of ions by electrospray ionisation (ESI, analyte concentration: 12.5 µM G-quadruplex, 0.5 mM KCl and 50 mM TMAA pH 6.8, solvent: acetonitrile-water, 1:1, capillary voltage: 4500 V, end plate offset voltage: 500 nebulizer gas pressure: 0.6/0.9 bar, dry gas flow rate: 6/9 l min^{-1}, dry temperature: 303 K) the desired ions were orthogonally deflected into the TIMS cell consisting of an entrance funnel and the TIMS analyser (carrier gas: N_2, temperature: 305 K, entrance pressure: 2.55 mbar, exit pressure: 0.89 mbar, IMS imeX ramp end: 1.66 1/K0, IMS imeX ramp start: 0.51 1/K0). As a result, the ions are stationary trapped. After accumulation (accumulation time: 70 ms), a stepwise reduction of the electric field strength leads to a release of ion packages separated by their mobility. After a subsequent focussing, the separated ions are transferred to the TOF-analyser.[25,26,27]

The ion mobility K was directly calculated from the trapping electric field strength E and the velocity of the carrier gas stream v_g *via*

$$K = \frac{v_g}{E} = \frac{A}{U_{release} - U_{out}} \quad (1)$$

where A is a calibration constant (based on calibration standards), $U_{release}$ is the voltage at which the ions are released from the analyser and U_{out} is the voltage applied to the exit of the tube. The ion mobility is corrected to standard gas density *via*

$$K_0 = K \frac{P}{1013\ \mathrm{hPa}} \frac{237\ \mathrm{K}}{T} \quad (2)$$

to obtain the reduced mobility K_0, where P is the pressure and T is the temperature. By using the Mason-Schamp equation, the collisional cross-section Ω can be calculated:

$$\Omega = \frac{(18\pi)^{\frac{1}{2}}}{16} \frac{ze}{(k_B T)^{\frac{1}{2}}} \left[\frac{1}{\mu}\right]^{\frac{1}{2}} \frac{1}{K_0} \frac{1}{N_0} \quad (3)$$

where ze is the ion charge, k_B is the Boltzmann constant, μ is the reduced mass of analyte and carrier gas and N_0 is the number density of the neutral gas.[25,26,27]

For calibration of both the TIMS and TOF analysers, commercially available Agilent ESI tuning mix was used. The instrument was calibrated before each measurement, including each change in the ion mobility resolution mode (“imeX” settings: survey, detect or ultra).

8.3 Native ESI-MS of G_4L^RCu

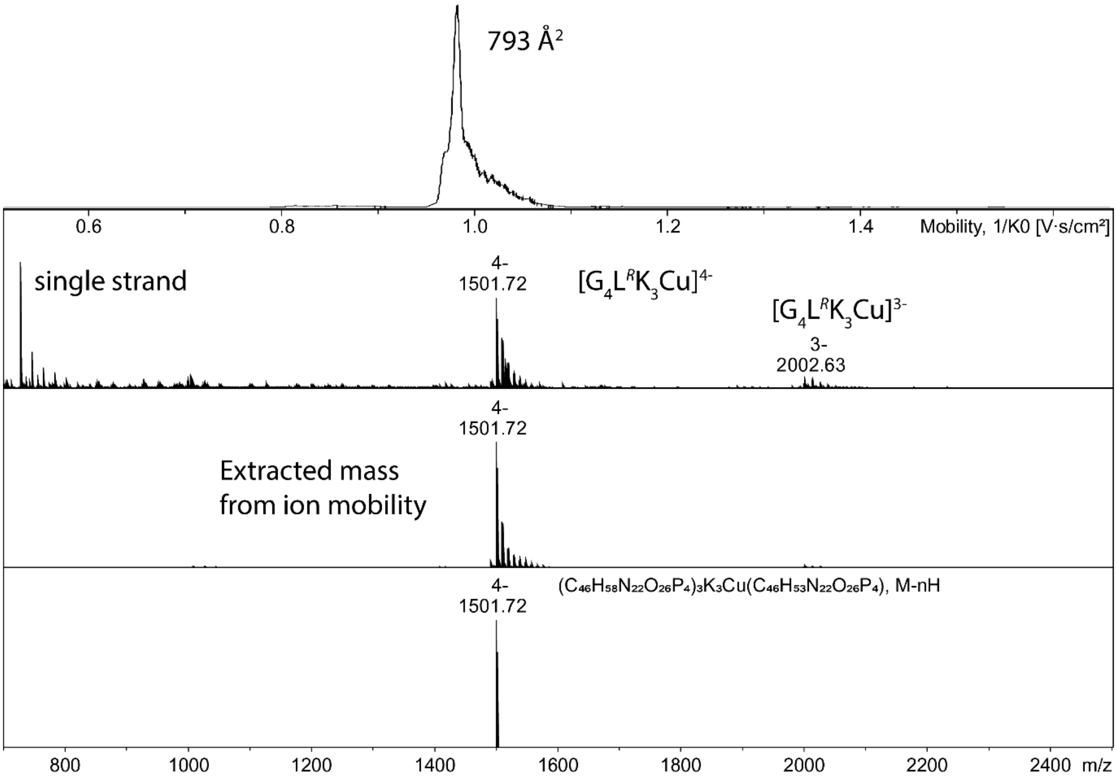

Figure 26. Native ESI-MS of G_4L^R in complex with Cu(II). To simulate the spectra 5 H^+ were subtracted to compensate the charges of Cu(II) and the three K^+ ions followed by additional 4 H^+ to simulate a 4^- charged species. In total 9 H^+ were subtracted.

8.4 Native ESI-MS of G_5L^R

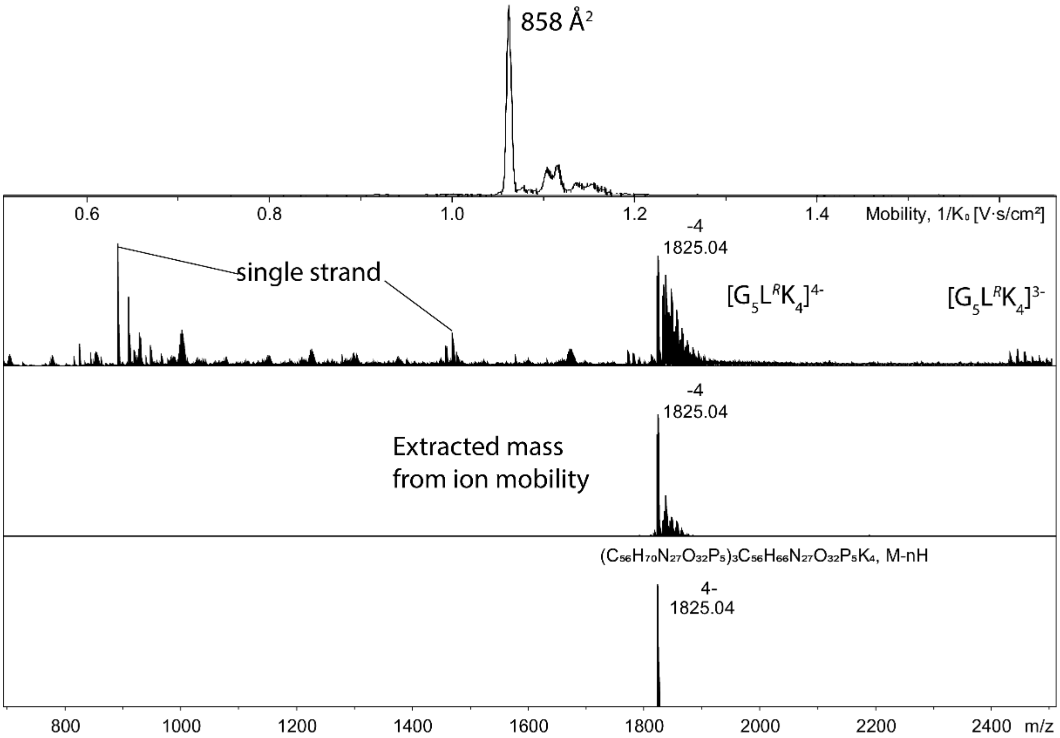

Figure 27. Native ESI-MS of G_5L^R. To simulate the spectra 4 H^+ were subtracted to compensate the charge of the four K^+ ions followed by additional 4 H^+ to simulate a 4^- charged species. In total 8 H^+ were subtracted.

8.5 Native ESI-MS of G_5L^RCu

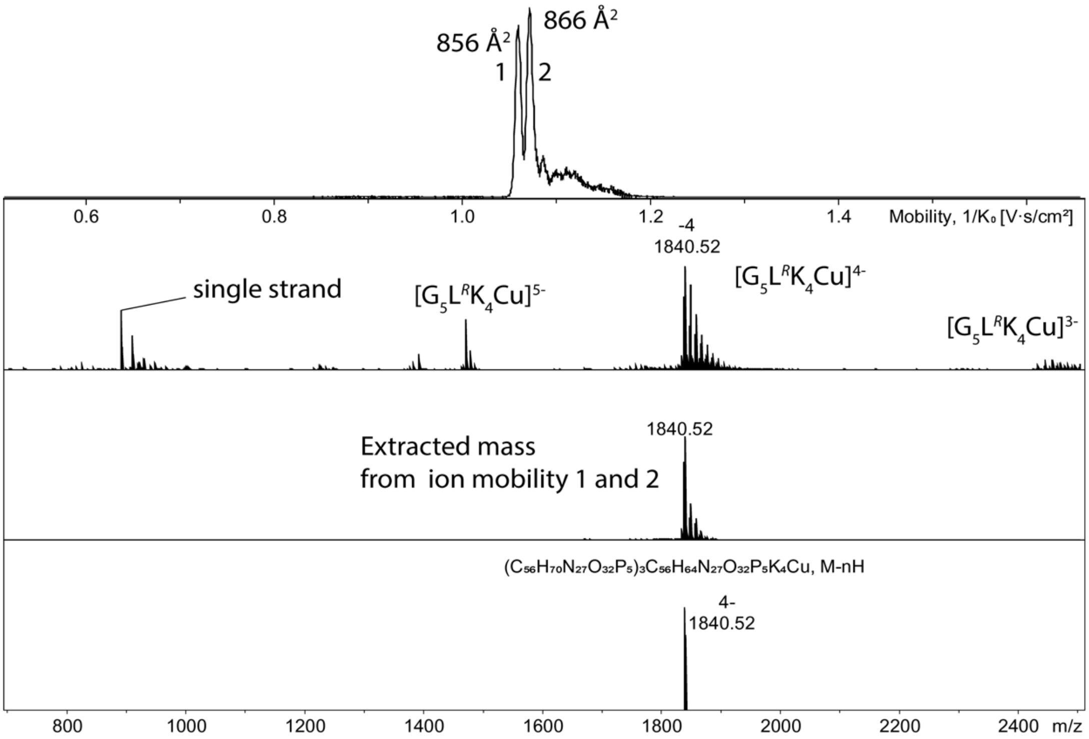

Figure 28. Native ESI-MS of G_5L^R. To simulate the spectra 6 H^+ were subtracted to compensate the charge of Cu(II) and the four K^+ ions followed by additional 4 H^+ to simulate a 4^- charged species. In total 10 H^+ were subtracted.

8.6 Native ESI-MS of G_5L^S

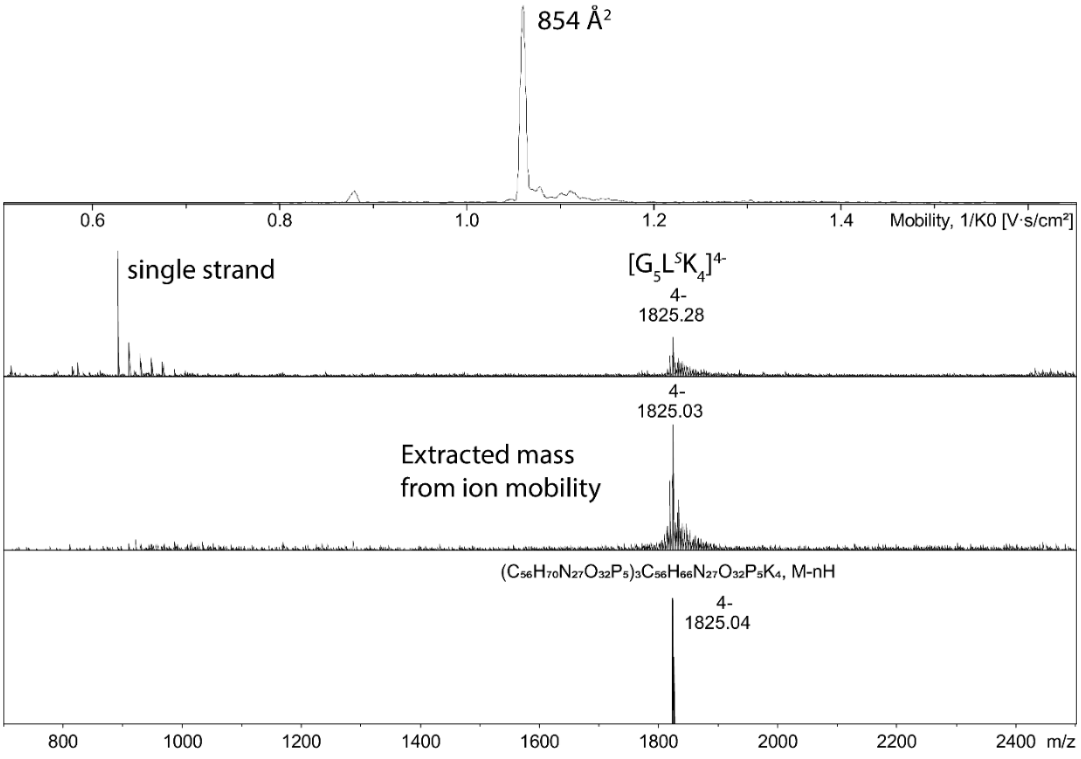

Figure 29. Native ESI-MS of G_5L^S. To simulate the spectra 4 H^+ were subtracted to compensate the charge of the four K^+ ions followed by additional 4 H^+ to simulate a 4^- charged species. In total 8 H^+ were subtracted.

8.7 Native ESI-MS of G_5L^SCu

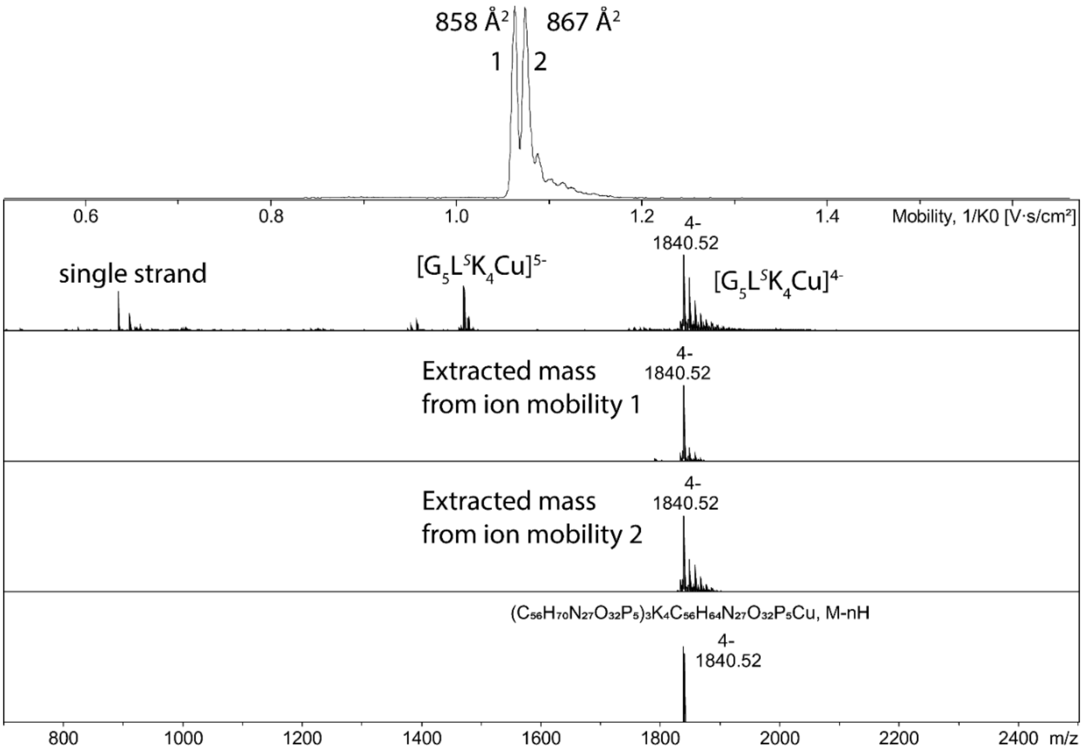

Figure 30. Native ESI-MS of G_5L^S in complex with Cu(II). To simulate the spectra 6 H^+ were subtracted to compensate the charge of Cu(II) and the three K^+ ions followed by additional 4 H^+ to simulated a 4^- charged species. In total 10 H^+ were subtracted.

9 Further analytical data

MALDI mass spectra (linear negative mode) were measured on a *Bruker Daltonics* MALDI-TOF mass spectrometer using a 3-HPA matrix (200 µL 5 mg/mL 3-HPA in 50:50 (v/v) acetonitrile/H_2O mixed with 12.5 µL 50 mg mL^{-1} diammonium hydrogen citrate in water) and *Bruker* recommended procedures. Calibration was achieved using *Bruker Daltonics* calibration kits.

The purity of DNA samples was checked by analytical reversed phase HPLC on *Agilent Technologies 1260 Infinity I or II* HPLCs equipped with *Agilent Zorbax 300 SB-C18 4.5 x 250 mm* columns. The flow rate was set to 1 mL min^{-1}. Prior to purification samples were filtered through *VWR Centrifugal filters* to ensure clear solutions and the column was equilibrated against buffer A. After equilibration samples were analysed using a standard gradient starting with buffer A going to buffer B over 30 min (see Table 2). Typical sample volumes were 10 µL with DNA concentrations ranging from 100 to 200 µM.

Buffer A: Aqueous solution of 0.05 M TEAA adjusted to pH 7.

Buffer B: 30 % v/v TEAA pH 7 in 70% v/v ACN.

Buffer C: Ultra-pure water (Ω = 18.2).

Buffer D: ACN.

Table 3. Solvent gradient in analytical HPLC runs.

	Analytical HPLC run			
**Time / min**	**A / %**	**B / %**	**C / %**	**D / %**
0	100	0	0	0
30	20	80	0	0
35	0	100	0	0
40	0	100	0	0
41	100	0	0	0
45	100	0	0	0

9.1 Analytical data of G3L^R

Sequence (5'→3'):
LGG G

(*R*)-L[1]

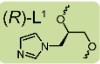

Analytical RP-HPLC

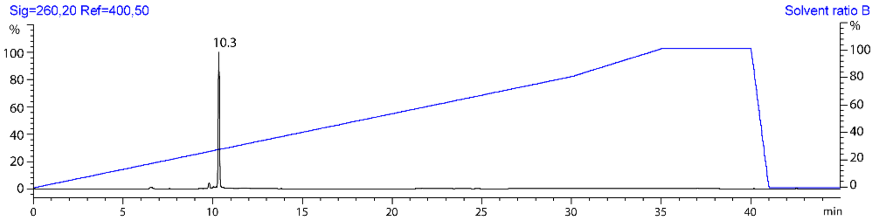

Maldi-Tof MS

mass (deconv.) = 11230.5
mass (calc.) = 1129.8 for $C_{36}H_{46}N_{17}O_{20}P_3$

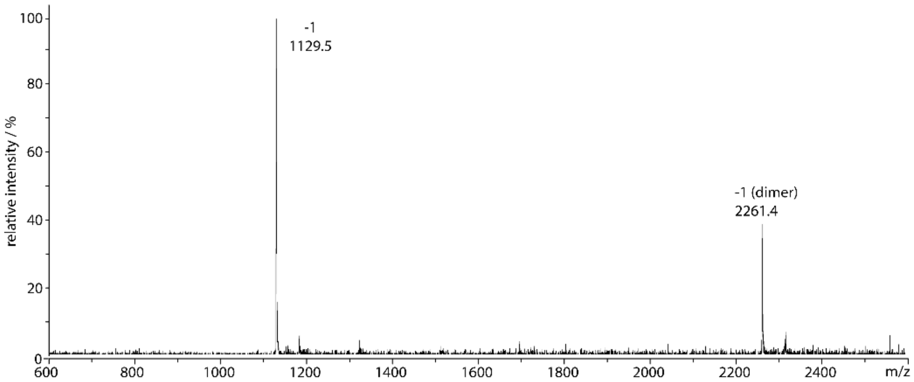

9.2 Analytical data of G3L^S

Sequence (5'→3'):
LGG G

(*S*)-L[1]

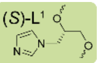

Analytical RP-HPLC

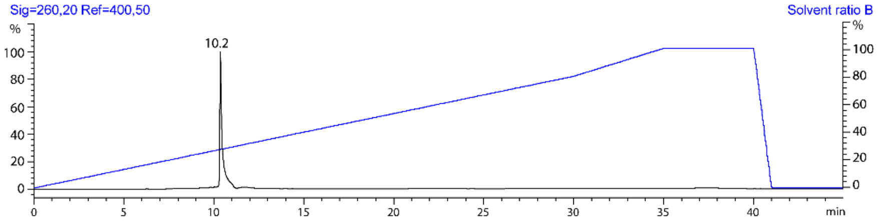

Maldi-Tof MS

mass (deconv.) = 1130.5
mass (calc.) = 1129.8 for $C_{36}H_{46}N_{17}O_{20}P_3$

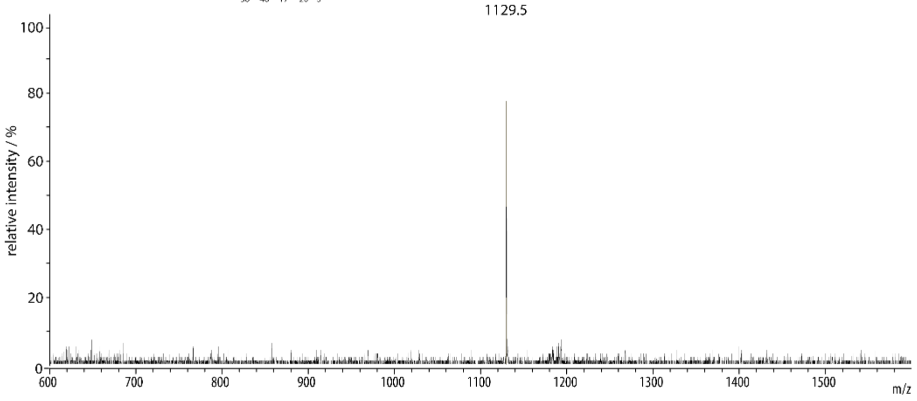

9.3 Analytical data of G4L^{R}

Sequence (5'→3'):
LGG GG

(*R*)-L[1]

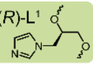

Analytical RP-HPLC

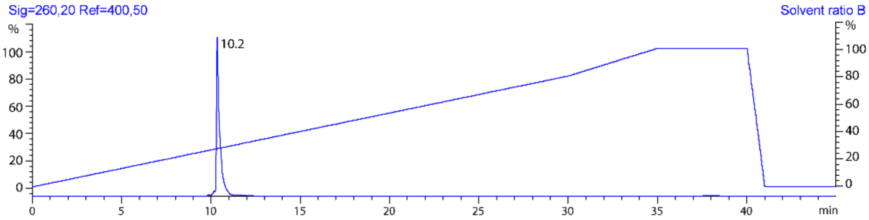

Maldi-Tof MS

mass (deconv.) = 1460.0
mass (calc.) = 1459.0 for $C_{46}H_{58}N_{22}O_{26}P_4$

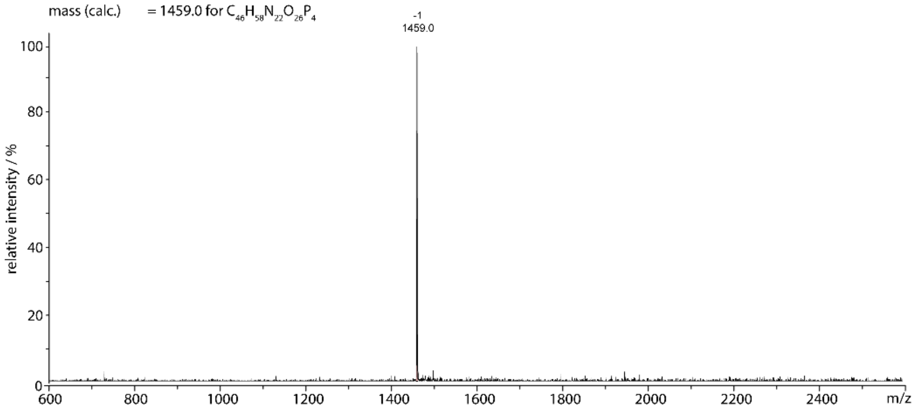

9.4 Analytical data of G4L^{S}

Sequence (5'→3'):
LGG GG

(*S*)-L[1]

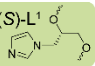

Analytical RP-HPLC

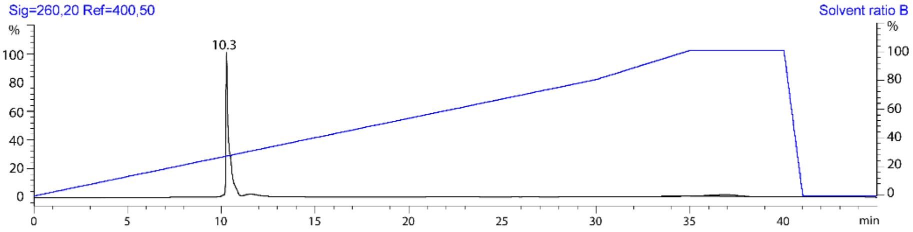

Maldi-Tof MS

mass (deconv.) = 1460.1
mass (calc.) = 1459.0 for $C_{46}H_{58}N_{22}O_{26}P_4$

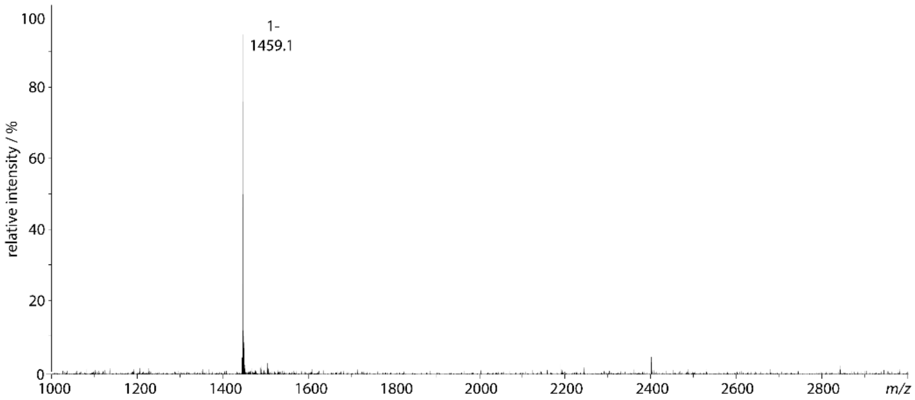

9.5 Analytical data of G_5L^R

Sequence (5'→3'):
LGG GGG

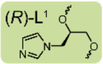

Analytical RP-HPLC

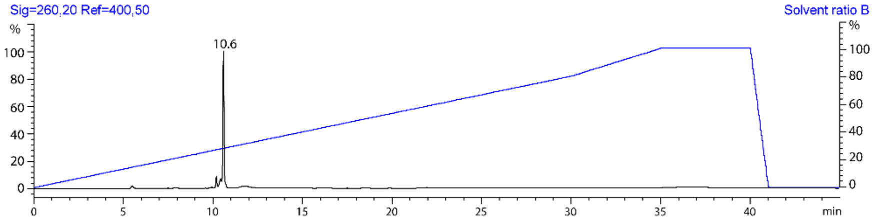

Maldi-Tof MS

mass (deconv.) = 1789.3
mass (calc.) = 1788.2 for $C_{56}H_{70}N_{27}O_{32}P_5$

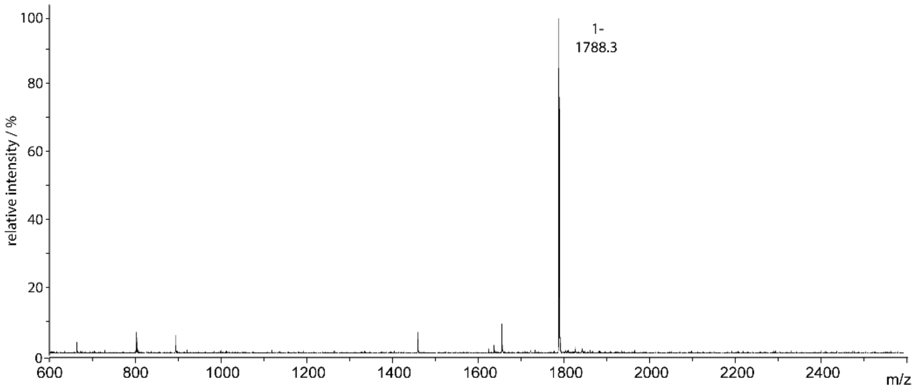

9.6 Analytical data of G_5L^S

Sequence (5'→3'):
LGG GGG

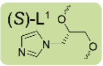

Analytical RP-HPLC

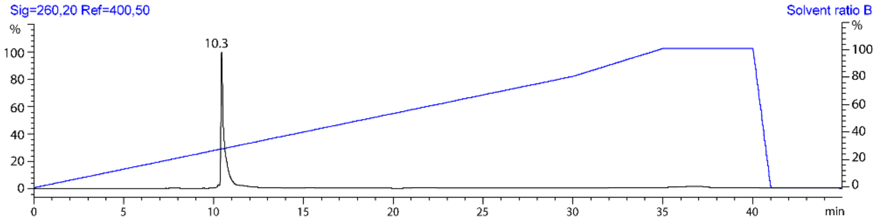

Maldi-Tof MS

mass (deconv.) = 1788.4
mass (calc.) = 1788.2 for $C_{56}H_{70}N_{27}O_{32}P_5$

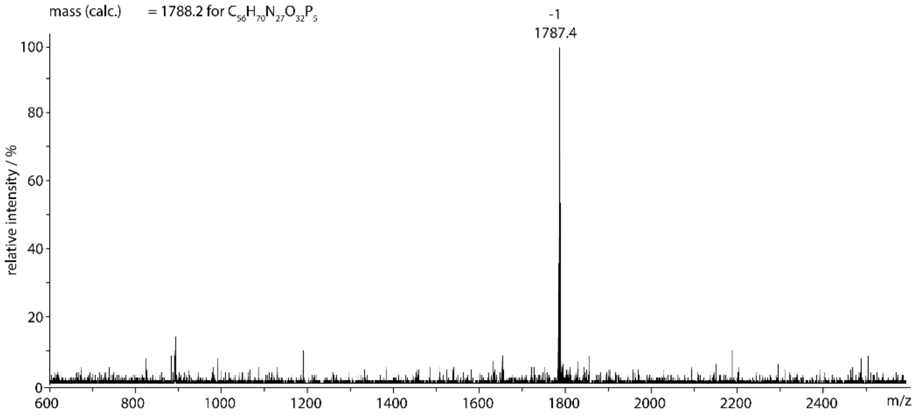

References

1 O. L. Acevedo, R. S. Andrews, *Tetrahedron Lett.,* 1996, **37**, 3931.
2 L. Zhang, A. E. Peritz, P. J. Carroll, E. Meggers, *Synthesis*, 2006, **4**, 645.
3 S. Pronk, S. Páll, R. Schulz, P. Larsson, P. Bjelkmar, R. Apostolov, M. R. Shirts, J. C. Smith, P. M. Kasson, D. van der Spoel, *Bioinformatics* 2013, **29**, 845.
4 D. van der Spoel, E. Lindahl, B. Hess, G. Groenhof, A. E. Mark, H. J. C. Berendsen, *J. Comput. Chem.,* 2005, **26**, 1701.
5 B. Hess, H. Bekker, H. J. C. Berendsen, J. G. E. M. Fraaije, *J. Comput. Chem.* 1997, **18**, 1463.
6 G. Bussi, D. Donadio, M. Parrinello, *J. Chem. Phys.,* 2007, **126**, 14101.
7 B. Hess, C. Kutzner, D. van der Spoel, E. Lindahl, *J. Chem. Theory Comput*., 2008, **4**, 435.
8 M. J. Abraham, T. Murtola, R. Schulz, S. Páll, J. C. Smith, B. Hess, E. Lindahl, *SoftwareX,* 2015, **1-2**, 19.
9 S. Páll, M. J. Abraham, C. Kutzner, B. Hess, E. Lindahl in *Lecture Notes in Computer Science* (Eds.: S. Markidis, E. Laure), Springer International Publishing, Cham, 2015.
10 D. M. Engelhard, J. Nowack and G. H. Clever, *Angew. Chem. Int. Ed*., 2017, **56**, 11640.
11 D. M. Engelhard, L. M. Stratmann, G. H. Clever, *Chem. Eur. J*., 2017, **24**, 2117.
12 D. M. Engelhard, A. Meyer, A. Berndhäuser, O. Schiemann and G. H. Clever, *Chem. Comm.*, 2018, **54**, 7455.
13 I. Ivani, P. D. Dans, A. Noy, A. Pérez, I. Faustino, A. Hospital, J. Walther, P. Andrio, R. Goñi, A. Balaceanu, *Nat. Methods,* 2016, **13**, 55.
14 A. Pérez, I. Marchán, D. Svozil, J. Šponer, T. E. Cheatham III, C. A. Laughton, M. Orozco, *Biophys. J.*, 2007, **92**, 3817.
15 M. J. Frisch, G. W. Trucks, H. B. Schlegel, G. E. Scuseria, M. A. Robb, J. R. Cheeseman, G. Scalmani, V. Barone, B. Mennucci, G. A. Petersson, *Gaussian 09, Revision D.01*, Gaussian, Inc., Wallingford CT, 2013.
16 S. Zheng, Q. Tang, J. He, S. Du, S. Xu, C. Wang, Y. Xu, F. Lin, *J. Chem. Inf. Model.* 2016, **56**, 811.
17 M. B. Peters, Y. Yang,B. Wang,L. Füsti-Molnár, M. N. Weave, K. M. Jr. Merz*, J. Chem. Theory. Comput.,* 2010, **6**, 2935.
18 C. Creze,B. Rinaldi,R. Haser, P. Bouvet,P. Gouet, *Acta Crystallogr.,Sect.D*, 2006, **63,** 682.
19 C. I. Bayly, P. Cieplak, W. Cornell, P. A. Kollman, *J. Phys. Chem.,* 1993, 97, 10269– 10280.
20 F.-Y. Dupradeau, A. Pigache, T. Zaffran, C. Savineau, R. Lelong, N. Grivel, D. Lelong, W. Rosanski, P. Cieplak, *Phys. Chem. Chem. Phys.,* 2010, **12**, 7821.
21 E. Vanquelef, S. Simon, G. Marquant, E. Garcia, G. Klimerak, J. C. Delepine, P. Cieplak, F.-Y. Dupradeau, *Nucleic Acids Res.,* 2011, **39**, W511.
22 F. Wang, J.-P. Becker, P. Cieplak, F.-Y. Dupradeau, R.E.D. Python: Object oriented programming for Amber force fields, Université de Picardie - Jules Verne, Sanford Burnham Prebys Medical Discovery Institute, 2013.
23 J.-L. Mergny, A. de Cian, A. Ghelab, B. Sacca, L. Lacroix, *Nucleic Acids Res.*, 2005, **33**, 81.
24 J. R. Wyatt, P. W. Davis and S. M. Freier, *Biochemistry*, 1996, **35**, 8002.
25 F. A. Fernandez-Lima, D. A. Kaplan, M. A. Park, *Rev. Sci. Instrum.* 2011, **82**, 126106.
26 D. R. Hernandez, J. D. DeBord, M. E. Ridgeway, D. A. Kaplan, M. A. Park, F. Fernandez-Lima, *Analyst,* 2014, **139**, 1913.
27 J.-F. Greisch, J. Chmela, M. E. Harding, D. Wunderlich, B. Schäfer, M. Ruben, W. Klopper, D. Schooss, M. M. Kappes, *Phys. Chem. Chem. Phys.* 2017, **19**, 6105.
28 R. Mera-Adasme, K. Sadeghian, D. Sundholm, C. Ochsenfeld, *J. Phys. Chem. B,* 2014, **118**, 13106.
